# Assessing underlying processes of implicit weight bias: a diffusion model analysis

**DOI:** 10.1038/s41598-026-67393-1

**Published:** 2026-08-22

**Authors:** Katja M. Pollak, Hanna Wachten, Julius Fenn, Raphael Hartmann, Constantin G. Meyer-Grant, Jana Strahler, Andrea Kiesel

**Affiliations:** 1 Department of Psychology, University of Freiburg, Engelbergerstrasse 41, Freiburg im Breisgau, 79106 Germany; 2 Department of Sports and Sports Science, University of Freiburg, Freiburg im Breisgau, Germany

**Keywords:** Weight bias, Implicit bias, Cognitive modeling, Response bias, Risk factors, Obesity, Computational science

## Abstract

Weight bias is a widespread issue, yet its cognitive mechanisms remain unclear. Using cognitive modeling across two studies ($N_1$ = 164, $N_2$ = 60), we investigated the processes underlying implicit weight bias. We assessed implicit weight bias with an Affective Priming-like Task, in which participants see a prime and then classify adjectives as positive or negative. Despite instructions to ignore the prime, responses are typically faster when prime and target share the same valence. In our studies, participants were primed with four gender-matched body shapes, of which we used the difference between the intermediate weight and higher weight primes to operationalize weight bias. To distinguish between response (starting point) and stimulus bias (drift rate), we applied a Bayesian Hierarchical Diffusion Decision Model. Results from both studies revealed an effect on the starting point: After intermediate weight primes, participants showed a stronger a priori tendency to choose the *positive* response compared with after higher weight primes. By contrast, drift rate differences between the primes were not credible. These findings suggest that implicit weight bias when measured with an Affective Priming-like Task manifests primarily as response, not as stimulus bias. This response bias is often linked to judgment-related rather than perceptual evidence-related processes and may inform the development of process-based anti-bias trainings.

Individuals with higher weight do not only face a range of physical challenges associated with excessive body weight but also often endure the harmful consequences of *weight bias*^1^. Weight bias, defined as the tendency to make unfair or irrational judgments about individuals based on their body weight, has been consistently linked to adverse mental and physical health outcomes^2–4^. For instance, several studies have demonstrated that individuals who experience weight bias report a reduced quality of life, increased symptoms of depression and anxiety^3^, and, paradoxically, greater weight gain compared to those who do not face such bias^5^. Moreover, patients with obesity receive poorer quality care from weight-biased health professionals^6^. Despite these profound consequences, research indicates that weight bias has remained relatively stable over time^7^, highlighting the importance of addressing this issue further.

## Theories explaining weight bias at a societal level

Several theories are discussed in the literature trying to explain weight bias, with the Attribution Theory^8^, the Social Identity Theory^9^, and the Socio-Cultural Theory^10^ being among the most prominent. According to Attribution Theory, weight bias arises when individuals attribute specific characteristics to a person’s weight, assuming that weight is entirely within a person’s control. This belief, despite evidence that body weight is influenced by complex biological and environmental interactions^11^, often leads to the perception of individuals with higher weight as being lazy and lacking willpower^12^. The Social Identity Theory^9,12^, in turn, posits that weight bias emerges as a result of group membership. Specifically, individuals who identify with a particular social group regarding body weight (e.g., people with *lower* weight) tend to show preferences for members of their own group while disadvantaging those from other groups (e.g., people with *higher* weight). Finally, the Socio-Cultural Theory^10,13^ suggests that weight bias stems from societal and cultural norms that prioritize the lower weight ideal while devaluing higher weight.

All of these theories receive empirical support and are therefore valuable for understanding different aspects of the development of weight bias. A commonality among these theories, however, is that they primarily characterize the origins and determinants of weight bias, while providing only broad and mostly vaguely specified accounts of the cognitive processes through which these influences operate at the individual level. Following Marr, this distinction can be understood in terms of different levels of theoretical analysis^14^. Specifically, the theories discussed above predominantly operate at the computational level, specifying which inputs are relevant for judgments and which biases they produce, but they do not explicitly formalize the algorithmic mechanisms by which these inputs are translated into biases.

Mathematical modeling offers an approach for addressing this limitation as it requires theoretical assumptions and cognitive processes to be specified explicitly rather than vaguely^15^. In the present research, we therefore focus on the algorithmic level of explanation, which has so far received little attention in the context of weight bias (for a similar approach on modeling data from an Implicit Association Test, see for example ref^16^.). By operating at a different level of analysis than the theories discussed above^14^, our goal is not to test these theories, but to complement them by providing a more fine-grained analysis of the cognitive processes underlying weight bias. To this end, we use the Diffusion Decision Model^17^, a theory-driven model of decision making, that enables the disentanglement of different cognitive processes and has therefore been proposed as valuable tool for decomposing cognitive biases^18,19^.

## The diffusion decision model

**Fig. 1:**
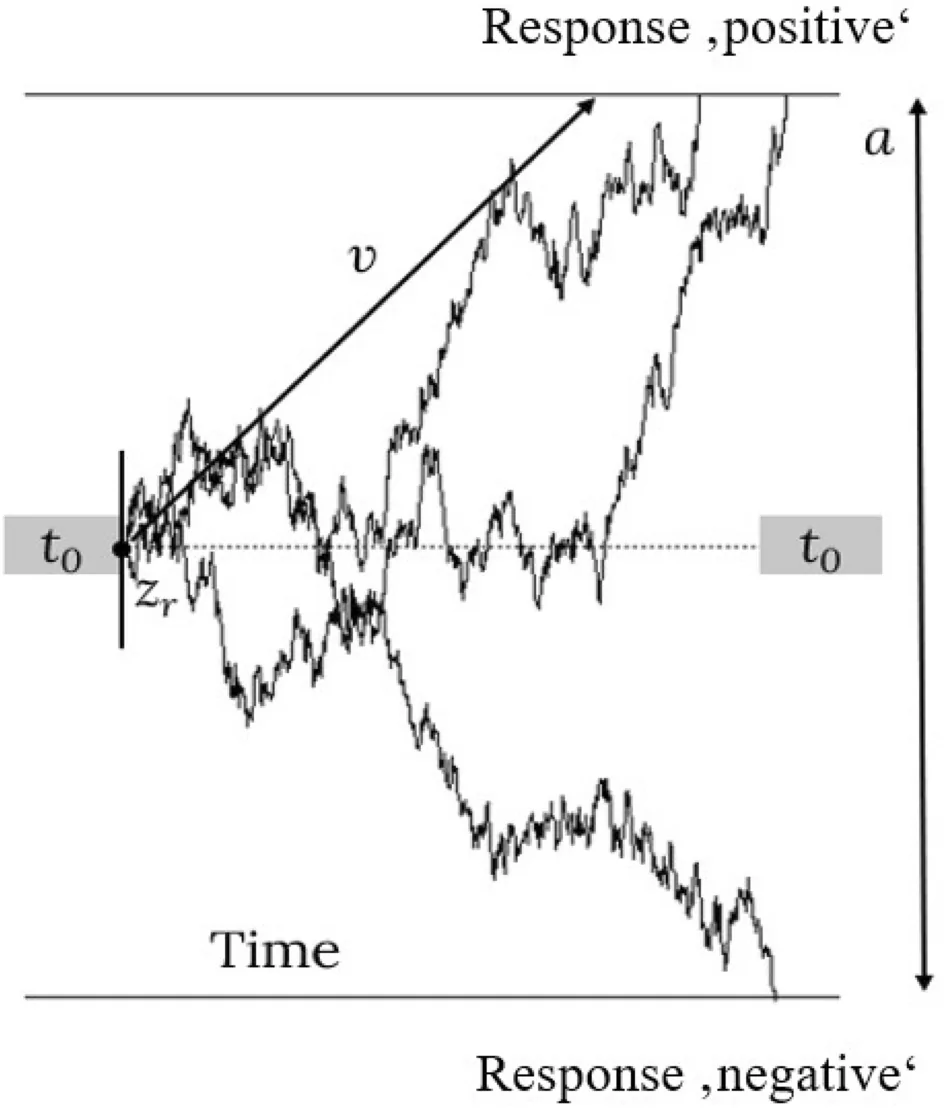
Illustration of the Diffusion Decision Model. *Note*. *v* = drift rate, *a* = boundary separation, $z_r$ = relative starting point, $t_0$ = non-decision time.

The Diffusion Decision Model (DM^17^) belongs to the class of evidence accumulation models, postulating that humans – when performing binary decisions – accumulate noisy evidence until one of two decision boundaries is reached; that is, either the one belonging to the first (the upper boundary) or the second (the lower boundary) response option (see Fig. 1). For example, when a person is asked to decide whether a presented word is *positive* or *negative*, they accumulate noisy evidence from the word, meaning that the word activates imperfect and uncertain information in memory (for example, ’this word is negative’), and these small pieces of information are gradually combined until a decision is reached. Once the decision is reached, the person then initiates the corresponding action (e.g., a button press)^20^. The DM comprises four main parameters that characterize the decision process, all of which are assumed to have a canonical interpretation: the drift rate *v*, the boundary separation *a*, the starting point *z*, and the non-decision time $t_0$^21^.

The first parameter, the drift rate (*v*), represents the rate at which individuals accumulate information towards the correct response. Higher drift rates thereby indicate faster information processing, which is typically associated with faster and more accurate responses (in Fig. 1, a higher drift rate would correspond to the arrow labeled with *v* being steeper). The threshold to which information is accumulated to, is captured in the second parameter, the boundary separation (*a*). More specifically, *a* reflects the level of response caution and is closely tied to the speed-accuracy trade-off. That is, a high boundary separation suggests a conservative response strategy, leading to slower but more accurate responses, whereas a low boundary separation implies a more liberal criterion, resulting in faster but less accurate responses (in Fig. 1, a higher boundary separation would be reflected by the two boundary lines being further apart). The relative starting point ($z_r$) indicates how much more evidence is needed to reach one boundary compared to the other, and thus can capture a potential a priori tendency toward one of the two response options. That is, whenever the starting point does not lie exactly in the middle between the two boundaries (relative starting point *≠ 0.5*) there is a response bias. If the respondent, for example, has a bias to respond with the right key (for example, because they are right-handed), the evidence accumulation process starts with a preference for the response option associated with that key. In Fig. 1, this would be reflected by a starting point that is closer to the boundary corresponding to the right-hand response option. Finally, the non-decision time ($t_0$) accounts for extra-decisional processes, such as perceptual encoding and response execution (the gray-shaded intervals in Fig. 1).

Overall, the DM is widely used in cognitive research due to its advantages over traditional analyses of reaction times (RTs) and accuracy rates. Unlike these traditional approaches, the DM provides a process-based explanation of how stimuli are evaluated, allowing researchers to link observed effects directly to underlying cognitive mechanisms^17^. Despite its strong theoretical foundation, the DM has, so far, only rarely been used in social cognition research. Recognizing its potential, Sherman *et al.* and Parker & Ramsey strongly advocate for its application in this field, emphasizing its ability to “identify the processes that best account for performance on the task of interest”^19^(p. 157)^22^. Furthermore, these model-derived process estimates can be leveraged to predict individual differences in behavior, potentially doing so “with greater specificity and acuity than raw implicit task performance”^19^ (p. 157) due to their process-pureness. In this way, modeling provides valuable insights into the cognitive foundations of social judgments and biases that can then be used to predict behavior.

## Localization of bias in the diffusion decision model

**Fig. 2:**
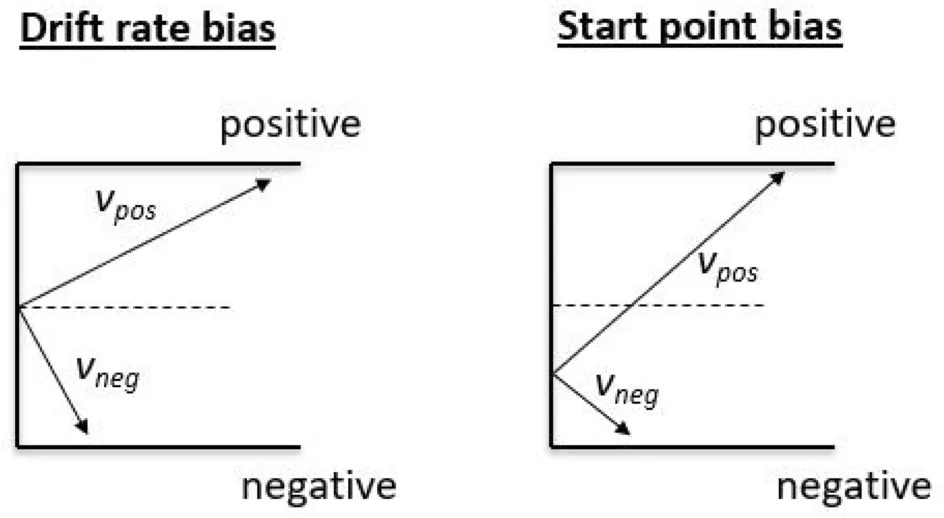
Illustration of Stimulus and Response Biases in the Diffusion Decision Model. *Note*. The drift rate [start point] bias refers to a stimulus [response] bias. In both cases, a bias towards the *negative* response is shown. For details, please see the main text. $v_{pos}$= drift rate for response option *positive*, $v_{neg}$= drift rate for response option *negative*.

To model weight bias within the DM framework, the model must be able to incorporate a bias into the decision-making process. The DM offers two ways of how to incorporate such a bias: either as a stimulus bias or as a response bias^18^. As shown in Fig. 2, the stimulus bias refers to a bias in drift rate, which affects the speed of evidence accumulation for the two response options (*positive* and *negative*). A negative stimulus bias means that evidence for the *negative* response is accumulated faster than for the *positive* response. This kind of bias is often interpreted as a biased perceptional processing because the processing of the target stimulus is biased (see e.g., ref^23,24^). In contrast, the response bias refers to a bias in starting point, which determines how much evidence is required for each response. A negative response bias indicates that the starting point is closer to the *negative* boundary, requiring less evidence to choose *negative* compared to *positive*. Importantly, such bias is an a priori bias in the sense that humans are *a priori* inclined to respond with the negative response option, without even having seen the target stimulus. The term *response bias* in the DM, hence, does not refer to a response to a stimulus (e.g., the prime), but to a bias toward one of the two *response options* before stimulus processing begins. Accordingly, this kind of bias is often interpreted as judgmental bias (see e.g., ref^23,24^). In summary, a stimulus bias leads to adjustments in how an object is processed, whereas a response bias leads to an a priori asymmetry in decision criteria^18^. Given that stimulus and response biases reflect different underlying mechanisms, distinguishing between them – as has already been done for racial bias^25^, for instance – is crucial to gain a deeper understanding of what drives weight bias and to design more effective anti-bias training programs.

## The present research

In the present research, we focus on participants’ *implicit *weight bias because behavioral data from tasks measuring implicit biases can be effectively modeled using the DM (e.g., ref^26^). Despite its advantages, particularly in identifying the underlying processes and in distinguishing between different kind of biases, no previous study has used the DM to investigate the cognitive processes associated with weight bias. In fact, to the best of our knowledge, the present studies are the first ones that use mathematical modeling to examine underlying processes in the field of weight stigmatization. Only through such modeling are we able to address our research question of whether weight bias is better characterized as a stimulus or a response bias.

## Methods

To address our research question, we analyzed data from two independent studies. The first dataset was drawn from a larger online study^1^, but we only included the reaction time task in the present analysis, as the other data – besides the sociodemographic data – were not relevant for our research question. This larger study was performed in accordance with the Declaration of Helsinki and received ethical approval from the ethics committee of the University of Freiburg (number 22–1375-S1). To assess the robustness of the findings, we analyzed pre-intervention data from a second study conducted in our laboratory, which was originally designed as an intervention to reduce weight bias. In the following, we refer to these datasets as Study 1 and Study 2.

### Participants

For Study 1, the final sample that we used for analysis consisted of *N* = 164 participants (124 females, 40 males) with a mean age of $\textit{M}_{\text {age}} = 26.15$ years ($\textit{SD} = 9.34$) and a mean BMI of $\textit{M}_{\text {BMI}} = 22.90$ ($\textit{SD} = 4.29$)^2^. Participants were not paid but instead had the chance to win one of ten 20€ vouchers. Students of the University of Freiburg could additionally receive course credits for their participation. Participants were recruited using convenience sampling through email advertisements via university mailing lists, flyer distribution in public spaces, and social media outreach.

For Study 2, the final sample that we used for analysis consisted of *N* = 60 participants (46 females, 14 males) with a mean age of $\textit{M}_{\text {age}} = 23.67$ years ($\textit{SD} = 5.23$) and a mean BMI of $\textit{M}_{\text {BMI}} = 22.40$ ($\textit{SD} = 3.16$)^2^. Participants either received course credits or €8 for their participation. Across both studies, eligibility criteria included a minimum age of 18 years, fluency in German, and for Study 1 additionally access to a computer or laptop with a keyboard. All participants provided informed consent prior to participation.

### Task & procedure

In both studies, to assess implicit attitudes toward different body shapes, we used a reaction time task analogous to the Affective Priming Task^27^. This task is designed to measure the automatic and relatively unconscious associations participants have with different primes by examining how quickly they categorize words following exposure to a certain prime. Typically, when the prime and the target word share the same evaluative valence (e.g., both positive or both negative), participants tend to respond more quickly and accurately, reflecting their implicit attitudes toward the prime category. Each trial of this task began with a fixation cross, displayed for a randomized duration between 1 000 and 1 700 ms. Next, a prime image was presented for 150 ms, followed by either a positive or negative German adjective (the target stimulus). Participants were instructed to ignore the prime and classify the word as quickly and accurately as possible as positive or negative by pressing one of two keys (key x or key m; the assignment to the *positive* and *negative* response was randomized between participants). The overall task structure of the two studies differed only in how non-responses within 2 000 ms were handled. In Study 1, the next trial started automatically after a 500 ms inter-trial interval when no response was given within the response window. In Study 2, participants were required to provide a response. If the response was made after 2 000 ms, participants received feedback, asking them to respond faster. Following this feedback, an inter-trial interval of 500 ms preceded the next trial, analogous to Study 1 (see Fig. S1 for an overview of the trial sequence in both studies).

**Fig. 3:**
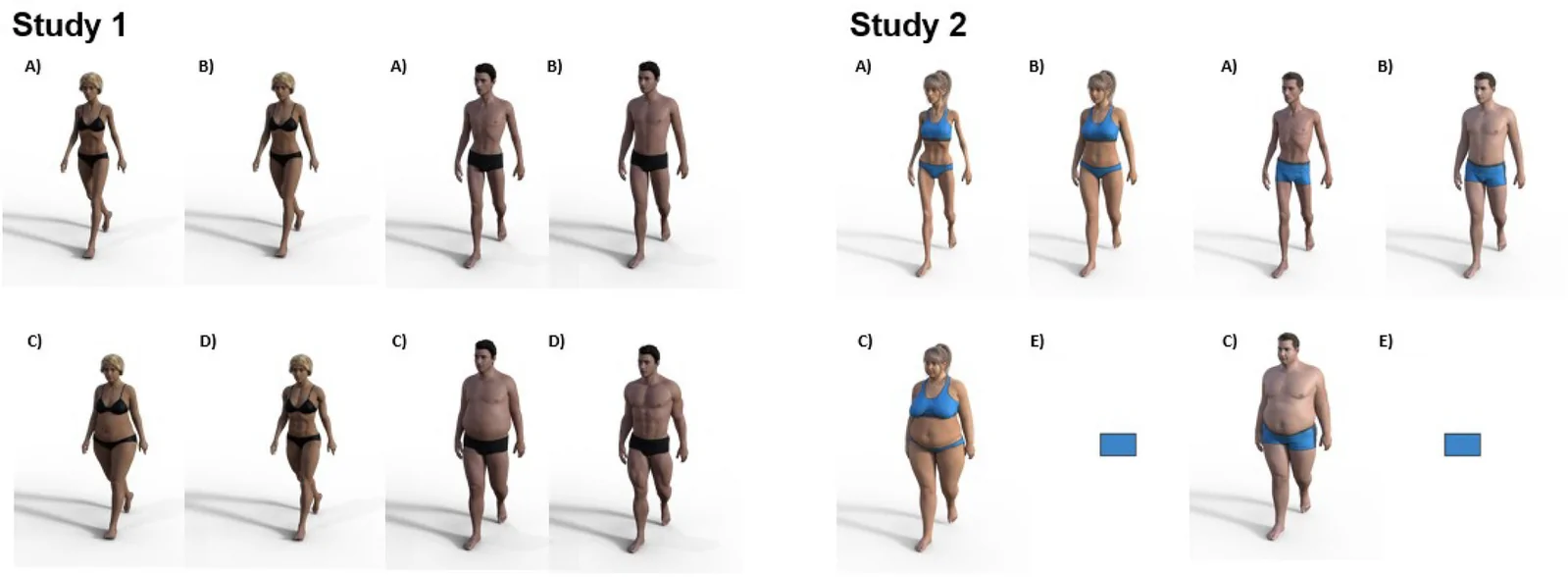
Primes used in both experiments. *Note*. A = lower weight, B = intermediate weight, C = higher weight, D = muscular, E = neutral.

In both studies, participants first completed 12 practice trials, that used facial primes to familiarize themselves with the task. The main experiment of Study 1 [Study 2] consisted of 288 [192] trials, divided into three [two] blocks, pairing four prime pictures with 24 adjectives that we have matched on arousal, absolute valence, number of letters, and frequency^3^, using the Berlin Affective Word List^28^ and the Aachen List of Trait Words^29^. The full list of adjectives can be found in the online supplementary material (see Table S1). In both studies, we used four distinct primes: lower weight, intermediate weight, higher weight, and muscular in Study 1, and lower weight, intermediate weight, higher weight, and neutral in Study 2 (see Fig. 3). We created all primes using DAZ Studio 3D^30^. In both studies, all participants were exposed to all prime-stimulus pairs in a randomized order with the only constraint that no target stimulus repeated itself in two adjacent trials. Because gender is one of the most salient categories in person perception^31^, we sought to minimize potential confounding effects of automatic gender categorization by matching the gender of the primes to participants’ gender in both studies. That is, male participants saw male primes and female participants saw female primes.

### Statistical analyses

Bayesian model fitting is a statistical approach that estimates model parameters by updating prior beliefs with observed data using Bayes’ theorem, yielding a posterior distribution that quantifies both the most likely parameter values and their associated uncertainty. A key advantage over frequentist methods is its ability to generate full posterior distributions, which explicitly represent parameter uncertainty while efficiently incorporating complex hierarchical structures. Within this Bayesian framework, hierarchical models offer several additional advantages. By means of partial pooling, extreme individual estimates are shrunk toward the group-level distribution, which increases the robustness of the fit to the sampling noise. Further, because the model explicitly represents both between-subject and within-subject variability, the resulting parameter estimates are more reliable and valid. Finally, the hierarchical structure allows the complex multilevel design of our experiment to be captured in a single coherent model while still providing a transparent quantification of parameter uncertainty^32–34^.

Therefore, for both studies, we implemented a Bayesian hierarchical version of the DM^34^ using Stan^35^. We let each parameter, namely drift rate (*v*), boundary separation (*a*), relative starting point ($z_r$), and non-decision time ($t_0$), vary across conditions (i.e., across different primes: lower weight, intermediate weight, higher weight, and muscular/neutral) and additionally estimated two drift rates; one for positive target stimuli and one for negative target stimuli. To keep the model as simple as possible, we only estimated the inter-trial variability of the non-decision time ($st_0$) but no other inter-trial variabilities (see Appendix Model Specification for details about the model specification).

As an inference criterion, we used the 95% highest density intervals (HDIs) for the group-level posteriors, which capture the most credible range of population-level parameter estimates. We defined weight bias as the difference between responses after the intermediate weight and the higher weight primes and considered this difference as credible when the 95% HDI of their posterior difference did not include zero. Regarding the drift rates, we compared their differences between the intermediate weight and the higher weight primes rather than the absolute drift rates for two reasons. First, the drift rate difference can be seen as an index on how processing differs for target stimuli with opposite valence; if weight bias is dependent on perceptual mechanisms, it should therefore manifest in that difference. And second, prior research indicates that evidence accumulation might generally be faster for positive words than for negative ones, regardless of the condition^36^. We therefore calculated the drift rate difference by subtracting the drift rate for the *negative* response from the drift rate for the *positive* response. Consequently, positive [negative] values of this difference indicate faster evidence accumulation for the *positive* [*negative*] response compared to the *negative* [*positive*] response. Regarding the starting point, since we used response coding, assigning the upper decision boundary to the *positive* response and the lower boundary to the *negative* response, we obtained starting point values between 0 and 1, with values higher [lower] than .5 indicating a bias towards the *positive* [*negative*] response. Consequently, we directly compared these values between the intermediate weight and the higher weight primes and additionally tested whether these values deviated from the unbiased decision of .5.

## Results

In Study 1 [Study 2], we excluded six [one] participants who had an accuracy rate of below 70% and additionally one [zero] participant who, upon visual inspection of their responses, had only guessed in the final block. On a trial level, we discarded responses with latencies lower than 300 ms and in Study 2 additionally those higher than 3 000 ms because we assume these responses to not come from the evidence accumulation process the DM postulates^37^. Additionally, in Study 1, we discarded those trials in which participants did not respond within the response deadline. In total, we excluded 374 trials (0.79 %) from the analysis in Study 1 and 4 trials (0.03 %) from the analysis in Study 2.

### Behavioral results

**Fig. 4:**
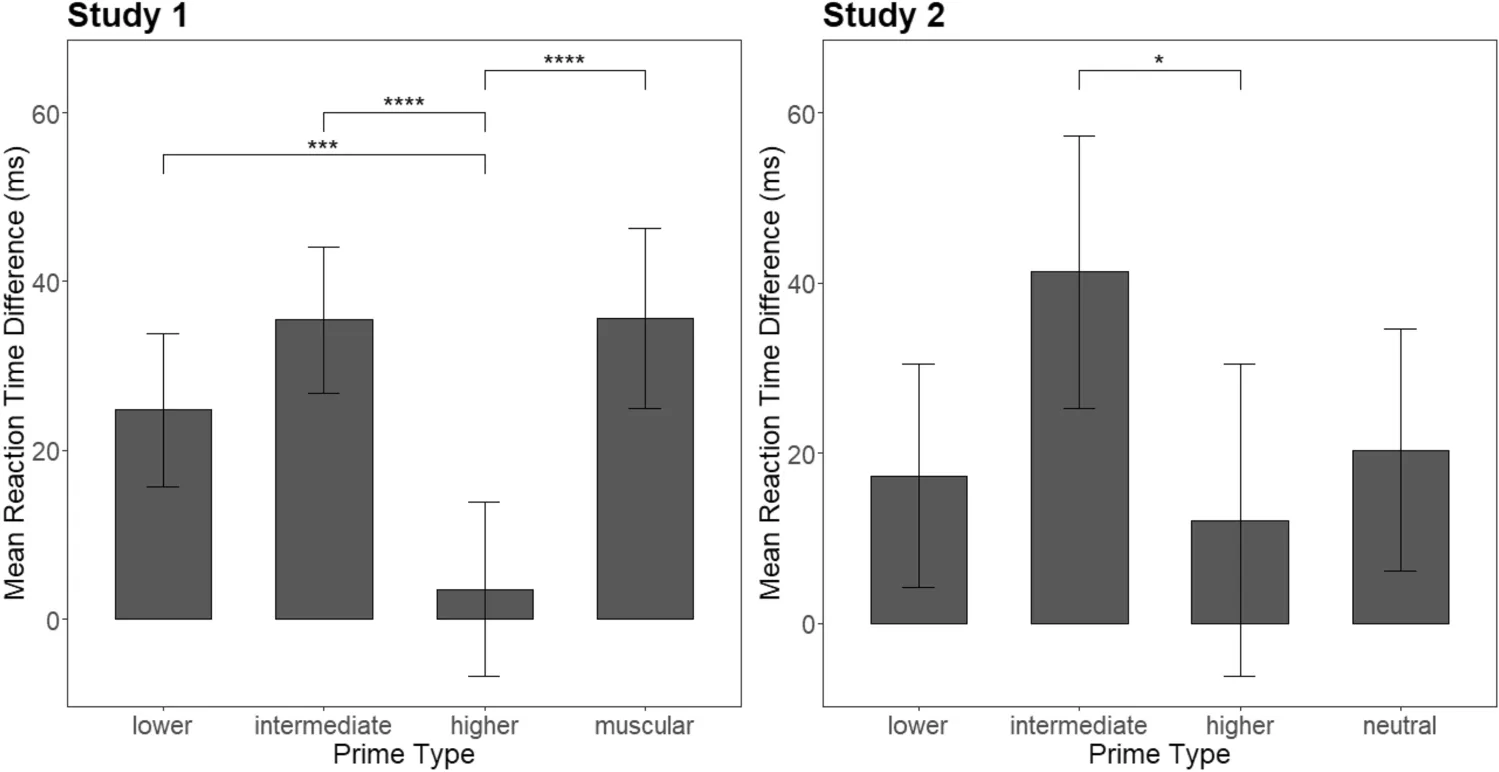
Mean response time difference scores between negative and positive Stimuli in both studies. *Note*. * *p* <.05 *** *p* <.001, **** *p* <.0001. Error bars indicate $M \pm 1.96 \times \text {standard errors of the mean}$, corresponding to a 95% confidence interval. Lower = lower weight; intermediate = intermediate weight; higher = higher weight.

Means and standard deviations of RTs and accuracy rates for both studies are displayed in Table 1. To analyze the behavioral data, we calculated mean RTs for correct responses per participant for both positive and negative target stimuli, separately for each prime. We then computed the difference between the mean RT for negative target stimuli and the mean RT for positive target stimuli, for each prime. These difference scores are presented in Fig. 4. In both studies, the one-way repeated measures ANOVA showed a main effect of prime type; Study 1: *F*(3, 489) = 16.98, *p* <.001, $\eta ^2_{\text {p}}$ =.094; Study 2: *F*(3, 177) = 3.29, *p* =.022, $\eta ^2_{\text {p}}$ =.053. For Study 1, a subsequent post-hoc t-test indicated that the mean RT difference following the higher weight prime was significantly smaller than the mean RT difference following the intermediate weight prime, *t*(163) = −6.50, *p* <.001, $d_z = -0.51$, 95% CI [*-0.67*, *-0.34*]. Similarly, in Study 2, a post-hoc t-test also indicated that the mean RT difference following the higher weight prime was significantly smaller than the mean RT difference following the intermediate weight prime, *t*(59) = −2.60, *p* =.012, $d_z = -0.34$, 95% CI [*-0.59*, *-0.07*].

**Table 1:** Descriptive results for response times and accuracy rates.

Measure	Stim	Lower	Intermediate	Higher	Muscular*/Neutral**
*Study 1*					
Response Time	Pos	710 (109)	702 (110)	729 (120)	707 (114)
	Neg	729 (118)	731 (120)	731 (121)	735 (117)
Accuracy Rate	Pos	92.7 (7.3)	93.1 (6.3)	90.4 (8.8)	93.1 (6.8)
	Neg	91.0 (8.8)	91.4 (8.4)	92.8 (7.3)	91.3 (8.4)
*Study 2*					
Response Time	Pos	671 (98)	650 (79)	676 (86)	664 (82)
	Neg	688 (83)	691 (87)	688 (95)	684 (89)
Accuracy Rate	Pos	92.0 (8.8)	92.2 (9.7)	90.5 (8.6)	92.2 (10.2)
	Neg	91.2 (7.1)	91.8 (7.2)	91.8 (7.0)	91.0 (7.3)

*in Study 1, ** in Study 2. Displayed are means (standard deviations) of correct trials only. Response time in ms, accuracy rate in %. Stim = stimulus valence; pos = positive; neg = negative; lower = lower weight; intermediate = intermediate weight; higher = higher weight.

### Modeling results

As suggested by Gelman *et al.*, we assessed model convergence and performed posterior predictive checks to ensure that the model fits the data appropriately^38^. The results of this can be found in the Appendix B.

**Fig. 5:**
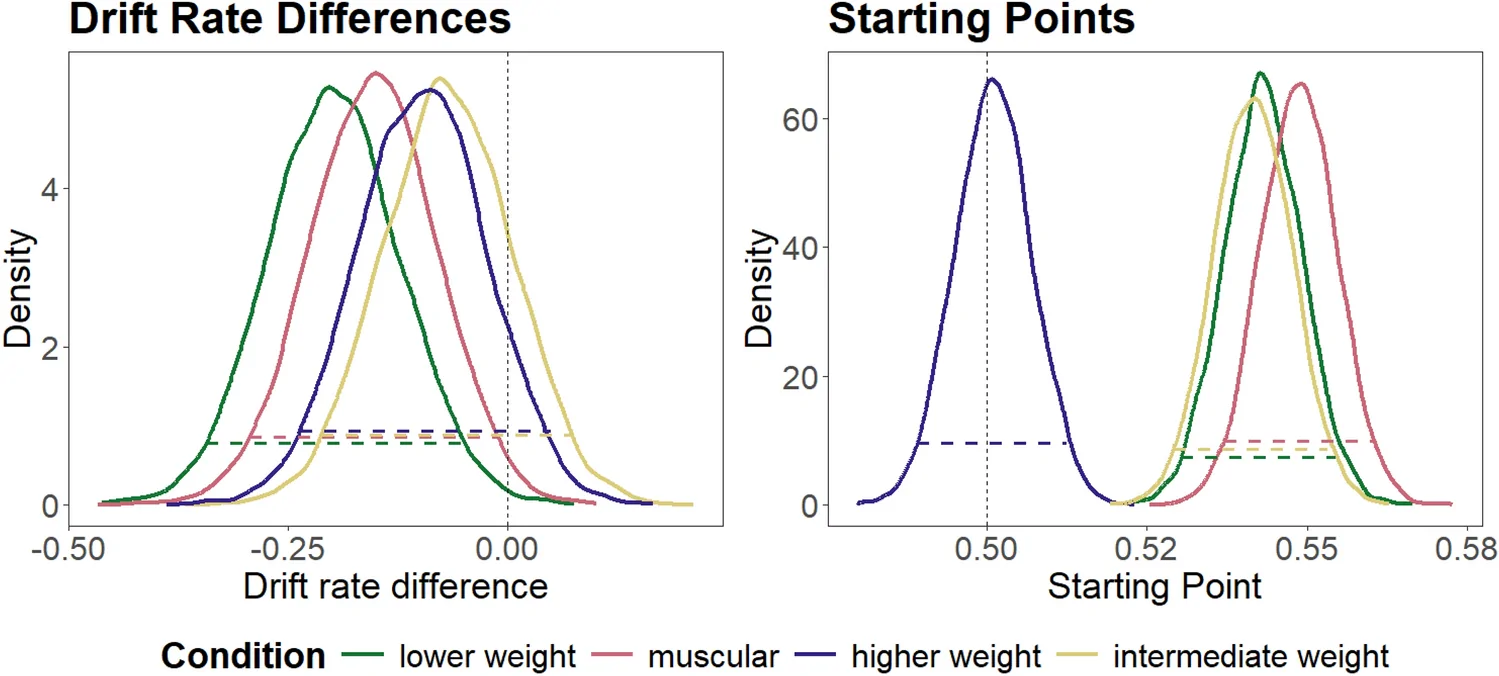
Posterior distributions of group-level parameters in Study 1. *Note*. Dashed lines indicate the 95% highest density intervals. See the online version for the color figure.

**Fig. 6:**
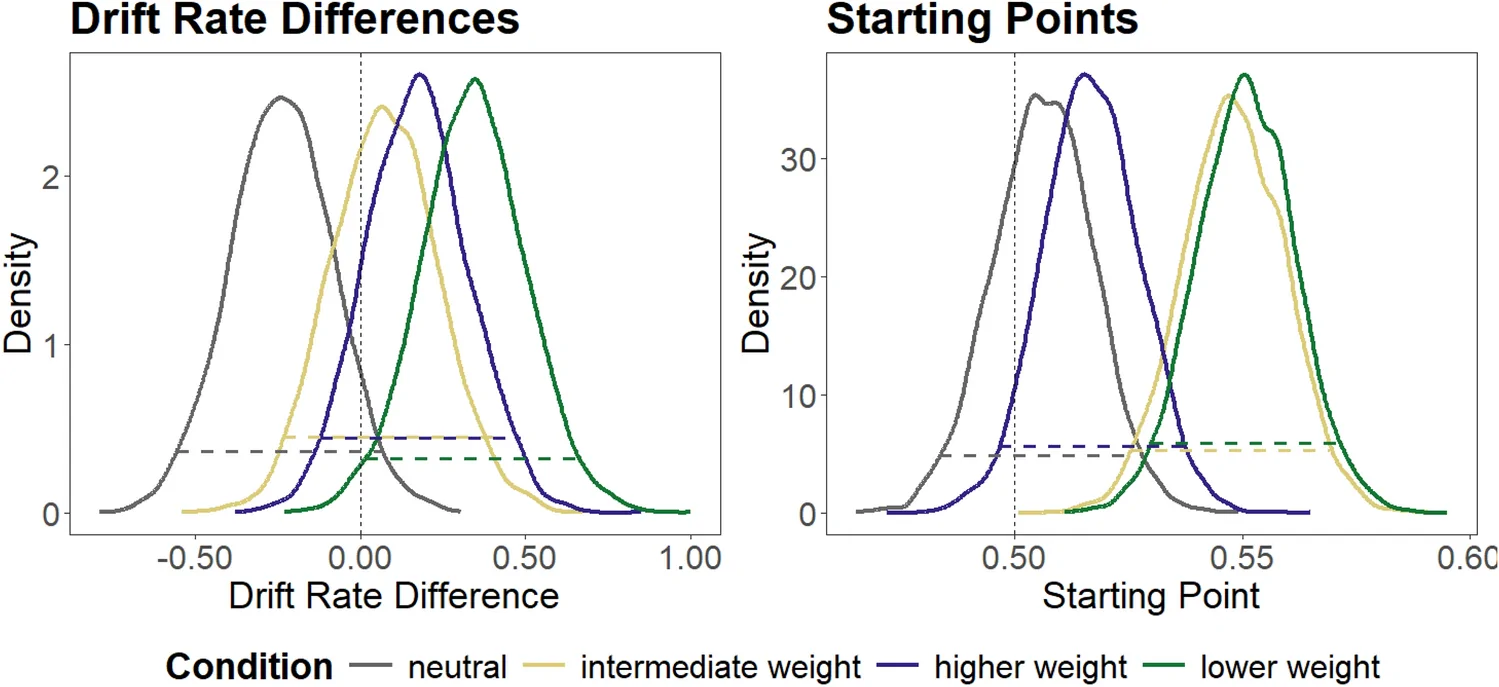
Posterior distributions of group-level parameters in Study 2. *Note*. Dashed lines indicate the 95% highest density intervals. See the online version for the color figure.

Regarding drift rate differences, the 95% HDIs of the posterior difference distributions in both studies included zero (Study 1 = [−0.22, 0.18], Study 2 = [−0.34, 0.54]), indicating no credible difference between the intermediate weight (Study 1: $\tilde{\mu }_{\text {intermediate weight}}$^4^ = −0.07, 95% HDI = [−0.21, 0.07]; Study 2: $\tilde{\mu }_{\text {intermediate weight}}$ = 0.07, 95% HDI = [−0.23, 0.40]) and the higher weight (Study 1: $\tilde{\mu }_{\text {higher weight}}$ = −0.10, 95% HDI = [−0.24, 0.05]; Study 2: $\tilde{\mu }_{\text {higher weight}}$ = 0.17, 95% HDI = [−0.12, 0.49]) primes (please see Fig. 5 and 6).

In contrast, in Study 1, we observed a credible difference in the starting point as the 95% HDI of the posterior difference distribution excluded zero ([−0.06, −0.02]). In Study 2, however, the two-sided 95% HDI included zero ([−0.06, 0.00]) but the one-sided did not [*-∞ , -0.00*]. Furthermore, 97.69 % of the posterior mass was below zero, providing strong directional evidence for a negative effect. That is, the 95% equal-tailed interval (another common type of credible interval^39^) excluded zero ([−0.06, −0.00]), supporting a credible difference between the intermediate weight and the higher weight prime. In both studies, we also observed the same qualitative pattern of results. That is, the starting points after seeing the higher weight prime (Study 1: $\tilde{\mu }_{\text {higher weight}}$ = 0.50, 95% HDI = [0.49, 0.51]; Study 2: $\tilde{\mu }_{\text {higher weight}}$ = 0.52, 95% HDI = [0.50, 0.54]) was lower than those following the intermediate weight primes (Study 1: $\tilde{\mu }_{\text {intermediate weight}}$ = 0.54, 95% HDI = [0.53, 0.55]; Study 2: $\tilde{\mu }_{\text {intermediate weight}}$ = 0.55, 95% HDI = [0.53, 0.57]).^5^ Additionally, in both studies, the relative starting points for the intermediate weight primes were credibly higher than 0.5 (see HDIs above), suggesting a general tendency to favor positive responses when exposed to these primes. The starting points after exposure to the higher weight prime, however, did not credibly differ from 0.5 (although it almost did in Study 2; please see Fig. 5 and 6)^6^, suggesting no general tendency to favor positive responses after seeing the higher weight primes. Interestingly, the starting points after seeing the muscular and lower weight primes were also credibly higher than 0.5, indicating pro-lower-weight and pro-muscular biases (Study 1: $\tilde{\mu }_{\text {lower weight}} = 0.54$, 95% HDI = [0.53, 0.55]; $\tilde{\mu }_{\text {muscular}} = 0.55$, 95% HDI = [0.54, 0.56]; Study 2: $\tilde{\mu }_{\text {lower weight}} = 0.55$, 95% HDI = [0.53, 0.57]).

## Discussion

We present two studies that provide the first formal, mathematical modeling account of data from an Affective Priming-like Task to investigate the cognitive mechanisms underlying weight bias. By applying the DM to reaction time data from such a task, we move beyond descriptive and correlational analyses and therefore offer a process-level account of implicit weight bias. Specifically, we assessed whether weight bias as measured with an Affective Priming-like Task reflects a stimulus (i.e., drift rate) or a response (i.e., starting point) bias within individuals. Our results showed in both studies the same qualitative pattern of results. That is, after seeing the intermediate weight prime, participants’ starting point was credibly closer to the *positive* threshold than after seeing the higher weight prime, suggesting a bias toward a more positive initial judgment after seeing the intermediate weight prime. Please note, however, that in Study 2 this effect was statistically less robust as it only emerged when testing one-sided or using the equal-tailed interval. All other model parameters remained unaffected by the type of prime, indicating that weight bias as measured with an Affective Priming-like Task neither influences the rate of evidence accumulation nor the non-decisional time nor the threshold separation but rather manifests as a response bias (i.e., a shift in the starting point) in the evidence accumulation framework.

A widely held belief has been that weight bias originates from an *anti-higher-weight* rather than a *pro-lower-weight* attitude. However, more recent studies have employed the Implicit Relational Assessment Procedure, which, unlike the Implicit Association Test (IAT), allows for a clearer distinction between these underlying attitudes. Findings from these studies suggest that the implicit weight bias may primarily stem from a *pro-lower-weight* rather than an *anti-higher-weight *attitude (e.g., ref^40,41^). This interpretation is also in line with broader research, indicating that lower weight body shapes are often idealized, and that muscularity is likewise a salient and socially valued body ideal (e.g., ref^42,43^). To examine whether this pattern is also reflected in our data, we compared the relative starting points to 0.5, which corresponds to unbiased decisions. Across our two studies, starting points were credibly above 0.5 for intermediate weight, lower weight, and muscular primes, indicating positive evaluations of these body shapes. In contrast, higher weight primes were the only body shape primes that were not credibly different from 0.5 and did not differ from the neutral stimulus. Thus, our findings are consistent with a *pro-intermediate-weight*, a *pro-lower-weight*, and a *pro-muscular* or, more broadly, a *pro-non-higher-weight* bias, suggesting that anti-higher-weight bias may manifest less as explicit negativity toward individuals with higher weight than as the absence of positivity afforded to them.

These findings support the notion that people might implicitly hold less positive/more negative attitudes toward individuals with higher compared to those with lower weight. Applying these results to social interactions, this response bias may reflect a pre-existing evaluative tendency toward a person with higher compared to a person with lower weight, emerging even before information from the interaction is available. Such early evaluative biases may have real-world consequences. Giel *et al*., for example, used images of individuals with varying BMIs and found that professionals in human resources exhibited a strong weight bias in hiring decisions^44^. Specifically, they were less likely to nominate individuals with obesity for supervisory positions and more likely to perceive them as unqualified for employment. Notably, these judgments were based solely on the individuals’ photographs^44^.

This evaluative tendency can stem from various sources. For example, it may arise from prior less positive/more negative experiences with individuals with higher weight, from stereotypical attributions such as perceiving them as lazy (as proposed by Attribution Theory^8^), from the derogation of out-group members (as postulated by Social Identity Theory^9^), or from less positive portrayals of individuals with higher weight in the media (as suggested by Socio-Cultural Theory^10^). Our approach does not contradict these perspectives but rather complements them by providing the algorithmic level of analysis^14^, i.e., insights into how such biases emerge within cognitive processes.

### Implications

Our findings may help in designing future anti-bias training programs for the broader society, as well as for decision-makers in human resources, healthcare, and clinical practice, who often disadvantage persons with higher weight in their decisions (e.g., ref^45–47^). Specifically, our results suggest that anti-bias training programs should focus on addressing the pre-existing evaluative tendencies, rather than perceptual processes. As mentioned earlier, all three theories outlined in this paper (Attribution Theory, Social Identity Theory, and Socio-Cultural Theory) can explain how such tendencies might arise. Therefore, these theories can serve as potential starting points for designing anti-bias training programs (as has been attempted, although not successfully in reducing implicit weight bias^48^). One particularly effective strategy, based on our results, could be to depict individuals with higher weight in positive contexts, just as lower weight and muscular individuals typically are. This could help ensure that people with higher weight are perceived as positively as those in other body categories, thereby weakening initial biases over time.

### Limitations and future research

We are aware that we applied a highly standardized task to assess weight bias and to enable Bayesian Hierarchical modeling, which poses certain potential limitations: First, as is typical in Bayesian Hierarchical modeling, we assumed a single population distribution from which all participants are drawn and are shrunk towards the population mean. However, it is possible that distinct clusters exist at the population level, such as one cluster representing participants with an implicit bias and another capturing those who do not show a bias (see e.g., ref^49^). Supporting this hypothesis, Brewis & Wutich found, for example, that a sample of women in Paraguay showed no implicit weight bias^50^. Despite this, we refrained from sampling a heterogeneous population due to our methodological focus and research objective. We therefore do not expect different clusters to be at play here. Nevertheless, future research sampling a more diverse group, including, for example, individuals with different cultural backgrounds (see e.g., ref^51^ for cultural differences in weight preferences), should focus on such qualitative interindividual differences, as this could help identify those who would benefit most from anti-bias trainings.

Second, although DM parameters are commonly interpreted as reflecting distinct cognitive processes, such mappings are not always straightforward (see e.g., ref^52^). In particular, in the standard interpretation of the DM, starting point biases reflect judgment-related rather than stimulus-related processes. However, recent research has shown that experimental manipulations of perceptual encoding designed to affect non-decisional processes, for instance, can also influence starting point estimates, highlighting the need for caution when interpreting model parameters as being reflective of specific cognitive processes^53^.

And third, we focused on applying the DM but did not use other modeling techniques such as Process Dissociation Models or the Quad Model which are viable alternatives (for further details, see ref^19^). Each model offers unique insights into object processing from different perspectives. Both, the Process Dissociation Models^54^ and the Quad Model^55^, for instance, disentangle automatic and controlled processes, which could also be relevant for studying social attitudes^56^. We chose the DM for its ability to differentiate between response and stimulus biases, yet we consider our research an initial step in applying statistical modeling to this field. Therefore, we urge future research to consider applying other statistical models such as the Quad model as well.

With respect to other *measures* of implicit weight bias, as suggested by a reviewer, future research should examine whether the distinction between stimulus and response biases generalizes beyond the Affective Priming-like Task used here. This would help to determine which aspects of the present findings generalize across tasks and which are specific to the task employed in our studies. The Weight IAT is a particularly relevant task, given its widespread use for assessing implicit weight bias^57^. However, a straightforward application of the DM to distinguish between stimulus and response bias in IAT data appears problematic for two reasons. First, by definition, the starting point in the DM is set *before* target onset. Unlike Affective Priming Tasks, however, the IAT does not include a preceding prime that could shift this pre-stimulus starting point. Second, the IAT response boundaries would represent compound categories: in compatible blocks, they would correspond to *thin-positive* and *fat-negative*^7^, whereas in incompatible blocks, they would correspond to *thin-negative* and *fat-positive*. Due to these compound response boundaries, a shift in the starting point would be difficult to interpret consistently as weight bias. Specifically, in compatible blocks, either direction (towards *thin-positive* or *fat-negative*) would be consistent with a weight bias, whereas in incompatible blocks, neither direction (towards *thin-negative* or *fat-positive*) would be consistent with a weight bias. In line with this, studies that apply diffusion modeling to IAT data typically fix the relative starting point at $z_r = a/2$ (see e.g., ref^16,59^). Future research should therefore investigate model extensions that allow stimulus and response biases to be distinguished in IAT data.

## Conclusion

In these two studies, we have, for the first time, demonstrated that formal, mathematical modeling, specifically the application of the Diffusion Decision Model, can provide meaningful insights into understanding weight bias. Our primary goal was to determine whether weight bias as measured with an Affective Priming-like Task manifests as a stimulus or a response bias, with the former often being associated with perceptual and the latter with judgmental processes. Our findings suggest that weight bias, measured with an Affective Priming-like Task and modeled within an evidence accumulation framework, is not characterized by a stimulus bias, as the processing of information does not appear to be altered but instead, it reflects an a priori response bias. Furthermore, our model indicates that participants show biases favoring non-higher-weight body shapes (i.e., lower weight, intermediate weight, and muscular), while no direct anti-higher-weight bias was observed. In light of these findings, we advocate for the continued use of the DM and other advanced statistical techniques to further deepen our understanding of weight bias and its impact on society, as well as to inform the design of effective anti-bias training programs.

## Supplementary Information


Supplementary Information.


## Figures and Tables

**Fig. 7:**
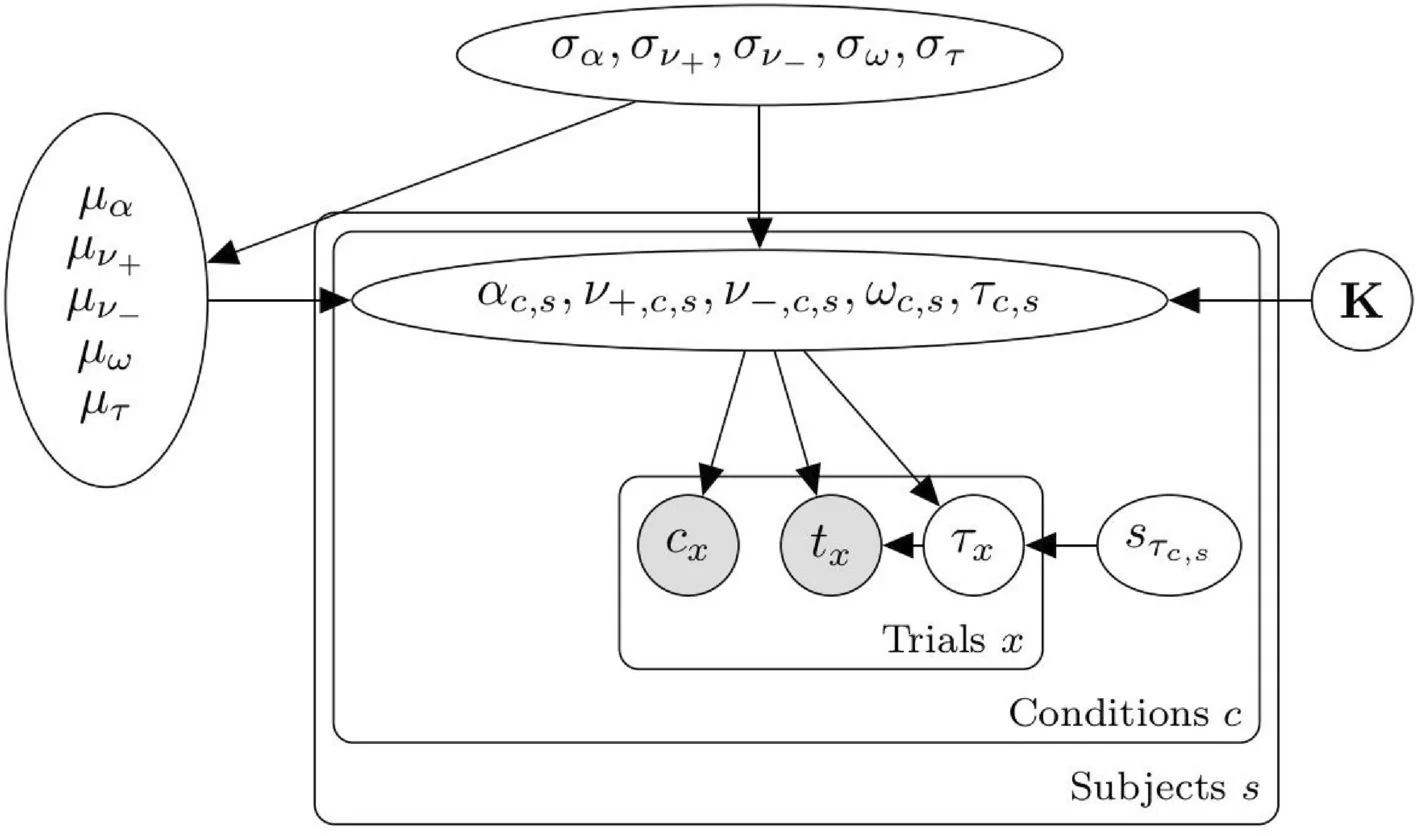
Directed acyclic graph of the model. *Note*. *x* = trials, *s* = subjects, *c* = conditions and {*+,-*} denote the valance of the stimulus. Gray colored circles/ellipses mark data (*c* denote categorical data and *t* the response times). All other circles/ellipses mark parameters of the model.

## Data Availability

All experimental code, analysis scripts, and raw data for the experiment are available on the associated OSF project (https://osf.io/3v7g8).

## Appendix A: Model specification

We assumed our data $Y_{t, \text {val}, \text {cnd}, \text {subj}}$ to be distributed as follows:

$$\begin{aligned} Y_{t, \text {val},\text {cnd}, \text {subj}} \sim \text {Wiener}(a_{\text {cnd},\text {subj}}, t0_{\text {cnd},\text {subj}}, w_{\text {cnd},\text {subj}}, v_{\text {cnd},\text {val},\text {subj}}), \end{aligned}$$

with *t* representing the trial, *val* the valance of the stimulus (i.e., a positive or negative word), *cnd* the condition (i.e., the prime), and *subj* the subject.

We adopted the latent-trait approach by Klauer, which models individual differences using a multivariate normal distribution of the five person-level parameters^60^. This approach requires that all model parameters are on a common domain (i.e., $\mathbb {R}$). Therefore the following transformations are applied:

A1 $$\begin{aligned} {\begin{matrix} \alpha _\text {cnd, subj} & = \ln (a_\text {cnd, subj})\\ \nu _{+,\text {cnd, subj}} & = v_{+,\text {cnd, subj}}\\ \nu _{-,\text {cnd, subj}} & = v_{-,\text {cnd, subj}}\\ \omega _\text {cnd, subj} & = \text {logit}(w_\text {cnd, subj})\\ \tau _\text {cnd, subj} & = \ln ({t_0}_\text {cnd, subj}) \end{matrix}} \end{aligned}$$

In the following we omit the indices for conditions and subjects to simplify the presentation.

On the common domain we chose a multivariate Gaussian distribution as a joint distribution for the transformed parameters above, such that

A2 $$\begin{aligned} \begin{pmatrix} \alpha \\ \nu _+\\ \nu _-\\ \omega \\ \tau \end{pmatrix} \sim \mathcal {N}(\vec {\mu }_\text {H}, \mathbf {\Sigma }), \end{aligned}$$

where $\vec {\mu }_\text {H}$ is the mean vector. $\mathbf {\Sigma }$ is the covariance matrix defined as

A3 $$\begin{aligned} \mathbf {\Sigma } = \left( \vec {s}\vec {\sigma }_\text {H}\right) ^T\times \textbf{I}_{P\times P} \times \textbf{K} \times \textbf{I}_{P\times P}\times \left( \vec {s}\vec {\sigma }_\text {H}\right) , \end{aligned}$$

where $\vec {s}$ is a vector of fixed (i.e., non-random) scaling factors, $\textbf{I}_{P\times P}$ is the identity matrix with dimensions being *P=5*, and $\textbf{K}$ is the correlation matrix of the five transformed parameters.

We used inter-trial variability for the non-decision time through a prior on the trial-wise non-decision times (per subject and condition) on the log-scale:

A4 $$\begin{aligned} {\begin{matrix} \tau _{tr} & \sim \mathcal {N}(\tau , \sigma _\tau )\\ \sigma _\tau & \sim \text {exp}(5). \end{matrix}} \end{aligned}$$

That is, the trial-wise non-decision times on the log-scale are sampled from a normal distribution with mean *τ* (subject-level non-decision time on log-scale per condition) and standard deviation from a rather uninformative exponential distribution (per subject and condition).

The hyper-priors are defined as follows:

A5 $$\begin{aligned} {\begin{matrix} \sigma _{\text {H},p}^2 & \sim \text {inv-}\chi ^2(\nu _{0,p}) \\ \mu _{\text {H},p} & \sim \mathcal {N}(\mu _{0,p}, \sigma _{0,p}^2 \sigma _{\text {H},p}^2) \\ \textbf{K} & \sim \text {LKJ}(\eta ), \end{matrix}} \end{aligned}$$

where *p* is the index for the *P=5* parameters, $\vec {\mu }_\text {H}=\left( \mu _{\text {H},\alpha }, \mu _{\text {H},\nu _+}, \mu _{\text {H},\nu _-}, \mu _{\text {H},\omega }, \mu _{\text {H},\tau }\right) ^{T}$ and $\vec {\sigma }_\text {H}=\left( \sigma _{\text {H},\alpha }, \sigma _{\text {H},\nu }, \sigma _{\text {H},\omega }, \sigma _{\text {H},\tau }\right) ^{T}$. The hyper-parameters are (rounded) $\vec {\nu }_0=\left( 4.12, 3.47, 3.47, 1.87, 2.35\right) ^{T}$, $\vec {\mu }_0=\left( 0.28, 1.83, 1.83, 0.0, -0.80\right) ^{T}$,^8^ and $\vec {\sigma }_0=\left( 0.58, 2.77, 2.77, 0.15, 0.26\right) ^{T}$ as well as $\vec {s}=\left( 0.34, 1.60, 1.60, 0.09, 0.15\right) ^{T}$. They are calibrated using the parameter collection of non-clinical data by Tran *et al *^61^ so that the individual-level parameters follow reasonable and mildly informative prior distributions. The degrees of freedom of the correlation matrix (*η*) is fixed to four.^9^ The model is depicted as a directed acyclic graph in Fig. 7.

To estimate the model, we used the No-U-Turn Sampler^62^ and ran four chains of 2 000 iterations each, with 1 000 additional burn-in iterations.

In order to examine the differences of the original model parameters between conditions, the transformed group-level parameters $\mu _{\text {H},\alpha }$, $\mu _{\text {H},\nu _+}$, $\mu _{\text {H},\nu _-}$, $\mu _{\text {H},\omega }$, $\mu _{\text {H},\tau }$ had to be transformed back to the original scale. Since the transformation was done on the subject-level, but we are interested on the group-level parameters $\mu _{\text {H},v_+}$ and $\mu _{\text {H},v_-}$, $\mu _{\text {H},a}$, $\mu _{\text {H},w}$, and $\mu _{\text {H},t_0}$, the following formulae were used:

A6 $$\begin{aligned} \mu _{\text {H},a}&= \exp \left( \mu _{\text {H},\alpha } + \frac{(\sigma ^2_{0,\alpha } + s_\alpha ^2 )\, \sigma ^2_{\text {H}, \alpha }}{2}\right) \end{aligned}$$

A7 $$\begin{aligned} \mu _{\text {H},v_+}&= \mu _{\text {H},\nu _+} \end{aligned}$$

A8 $$\begin{aligned} \mu _{\text {H},v_-}&= \mu _{\text {H},\nu _-} \end{aligned}$$

A9 $$\begin{aligned} \mu _{\text {H},w}&= \int _{-\infty }^{\infty } \frac{\tanh {\left( \frac{x}{2}\right) } + 1}{2 \sqrt{2 \pi (\sigma ^2_{0,\omega } + s_\omega ^2 )\, \sigma ^2_{\text {H}, \omega }}} \exp \left( \frac{-(x - \mu _{\text {H},\omega })^2}{2 (\sigma ^2_{0,\omega } + s_\omega ^2 )\, \sigma ^2_{\text {H}, \omega }}\right) \text {d}x \end{aligned}$$

A10 $$\begin{aligned} \mu _{\text {H},t_0}&= \exp \left( \mu _{\text {H},\tau } + \frac{(\sigma ^2_{0,\tau } + s_\tau ^2) \, \sigma ^2_{\text {H}, \tau } }{2}\right) . \end{aligned}$$

## Appendix B: Model convergence and posterior predictive checks

To ensure model convergence, we visibly inspected the Markov chains for each group-level parameter. All of them follow the desirable pattern of a caterpillar (please find the trace plots in Figure S2 and S8 in the SOM). Additionally, we assessed whether all chains have intermixed by relating the within-chain variance to the between-chain variance and calculating the $\hat{R}$ statistic for each group-level parameter^63^. All $\hat{R_s}$ were below the critical threshold of 1.01, indicating good convergence.

To examine the model fit we conducted posterior predictive checks for both studies. That is, for each study, for each MCMC iteration (8 000), we sampled data for each participant, calculated the accuracy rates for each participant and condition (stimulus and prime) and calculated the first to third quartiles of the RTs for each participant, condition (stimulus and prime) and response. Then, we averaged the values over participants and compared them to the corresponding observed accuracies and first to third quartiles of the RTs. The observed values should lie within the 8 000 generated values. Overall, the posterior predictive checks indicated a good model fit. Across both studies, the observed accuracy rates fell well within the distribution of the predicted accuracies. Similarly, the observed reaction time quartiles were largely covered by the model’s posterior predictions, with only few deviations. The corresponding graphical model fits are displayed in the supplemental material S3 - S6 and S9 - S12.

## Footnotes

1 see https://www.psycharchives.org/en/item/0aac4809-e06c-4de9-82c5-5dcf42b8332f for the preregistration of the larger study.

2 based on self-reported weight and height from *n* = 163 participants (Study 1) and *n* = 60 participants (Study 2).

3 all Cohen’s *d*s of differences between positive and negative words <.20.

4 We use $\tilde{\mu }$ to refer to the median of the respective posterior group-level distribution.

5 To make the difference in the starting point more concrete, we simulated data from the median parameter estimates, allowing only *w* to vary between intermediate and higher weight primes, while pooling the remaining parameters across these two conditions. The simulations yielded slightly faster *positive* responses (Study 1: 19.53 ms; Study 2: 14.87 ms), slightly slower *negative* responses (Study 1: -18.87 ms; Study 2: -13.96 ms), and a higher probability of *positive* responses after intermediate weight than after higher weight primes (Study 1: 1.65 percentage points; Study 2: 1.29 percentage points).

6 We also examined boundary separations and non-decision times in both studies but did not find differences between primes in either of these two parameters. The corresponding group-level posterior distributions are presented in Figure S7 and S13 in the SOM.

7 Please note that Weight IATs typically use the terms *thin* and *fat* although the American Psychological Association suggests the usage of more neutral terms^58^.
