# Supplementary material for: Assessing underlying processes of implicit weight bias: a diffusion model analysis
Source: Sci Rep. 2026 Aug 22;16:27408. doi: 10.1038/s41598-026-67393-1 (PMC13534490; doi:10.1038/s41598-026-67393-1)
Supplement: Supplementary file 1 [file 41598_2026_67393_MOESM1_ESM.pdf]

Supplemental Online Material for

Assessing underlying processes of weight bias: A diffusion model analyses

by Katja M. Pollak, Hanna Wachten, Julius Fenn, Raphael Hartmann, Constantin G. Meyer-Grant, Jana Strahler, Andrea Kiesel

Table S1

Target stimuli used in both studies

**Positive**	**Negative**
robust, heiter, autonom, lieb, agil, eifrig, munter, tapfer, wachsam, vital, loyal, spontan	schwach, mies, lustlos, gemein, trotzig, unfair, passiv, stur, labil, einsam, naiv, eitel

Figure S1

Trial Sequence of the Reaction Time Task used in both studies

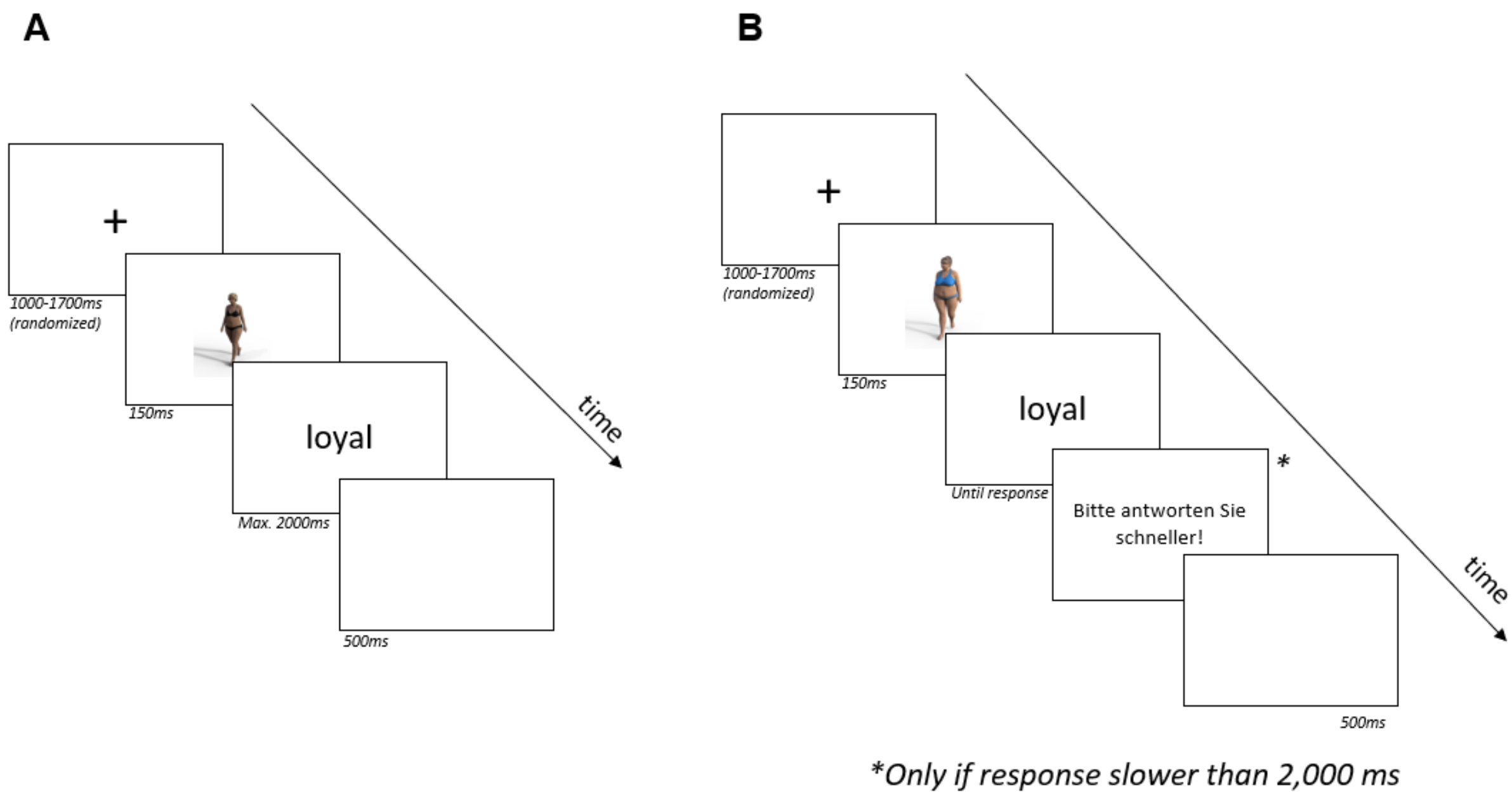

Note. A = Trial sequence in Study 1, and B = Trial sequence in Study 2. *Bitte antworten Sie schneller* is German for *Please respond faster*. Please see the text in the manuscript for more details on the task.

Figure S2

Trace Plots of all Group-Level Parameters for Study 1

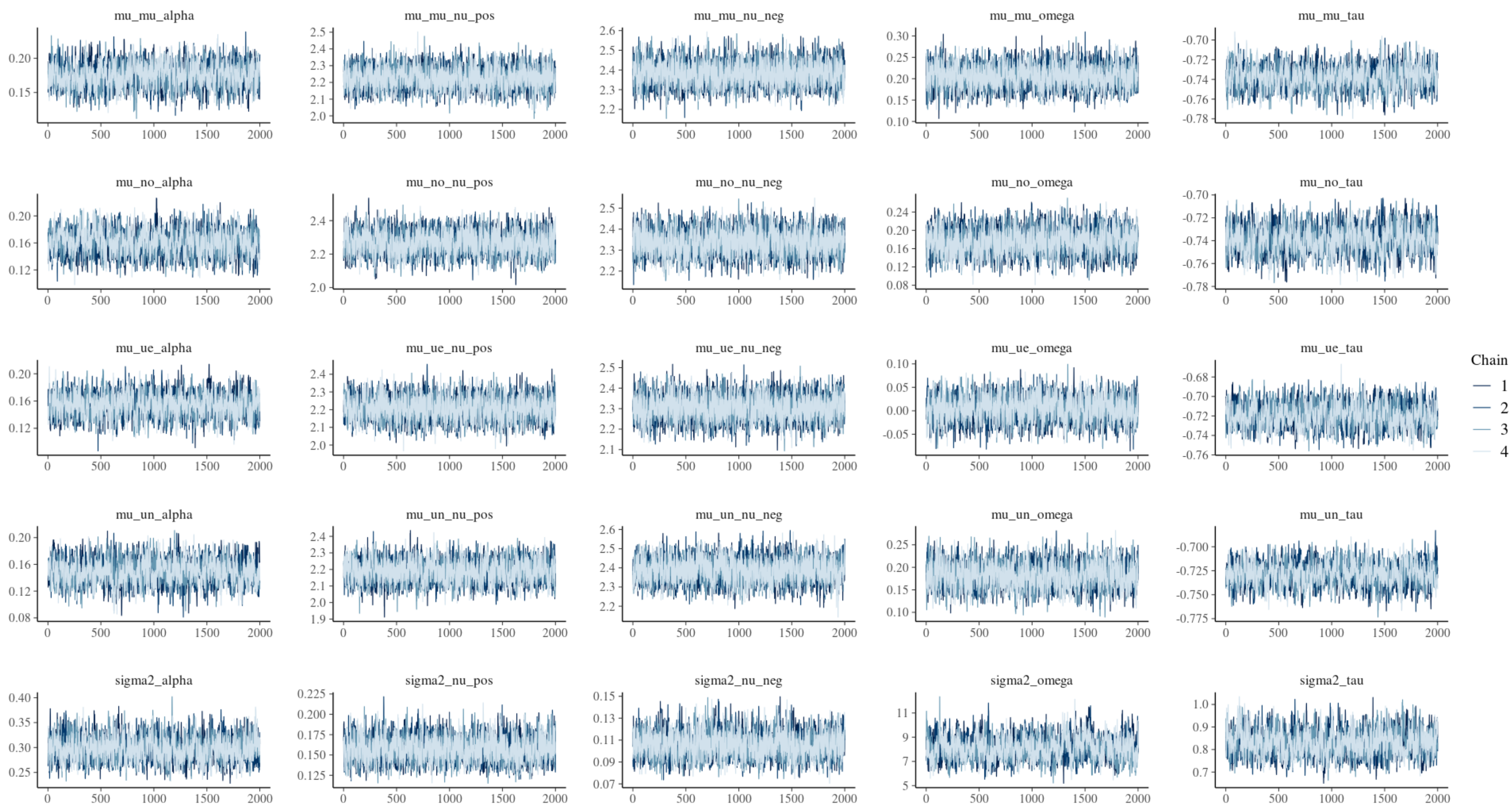

Note. The headings are structured as follows: the first part: mu = mean of group level distribution, sigma2 = variance, the second part (not for sigma): mu = muscular, no = intermediate weight, ue = higher weight, un = lower weight, the third part: alpha = boundary separation, nu = drift rate, omega = starting point, tau = non-decision time, the fourth part (only for drift rates): pos = positive, neg = negative.

Figure S3

Posterior Predictive of the Accuracy Rate for Study 1

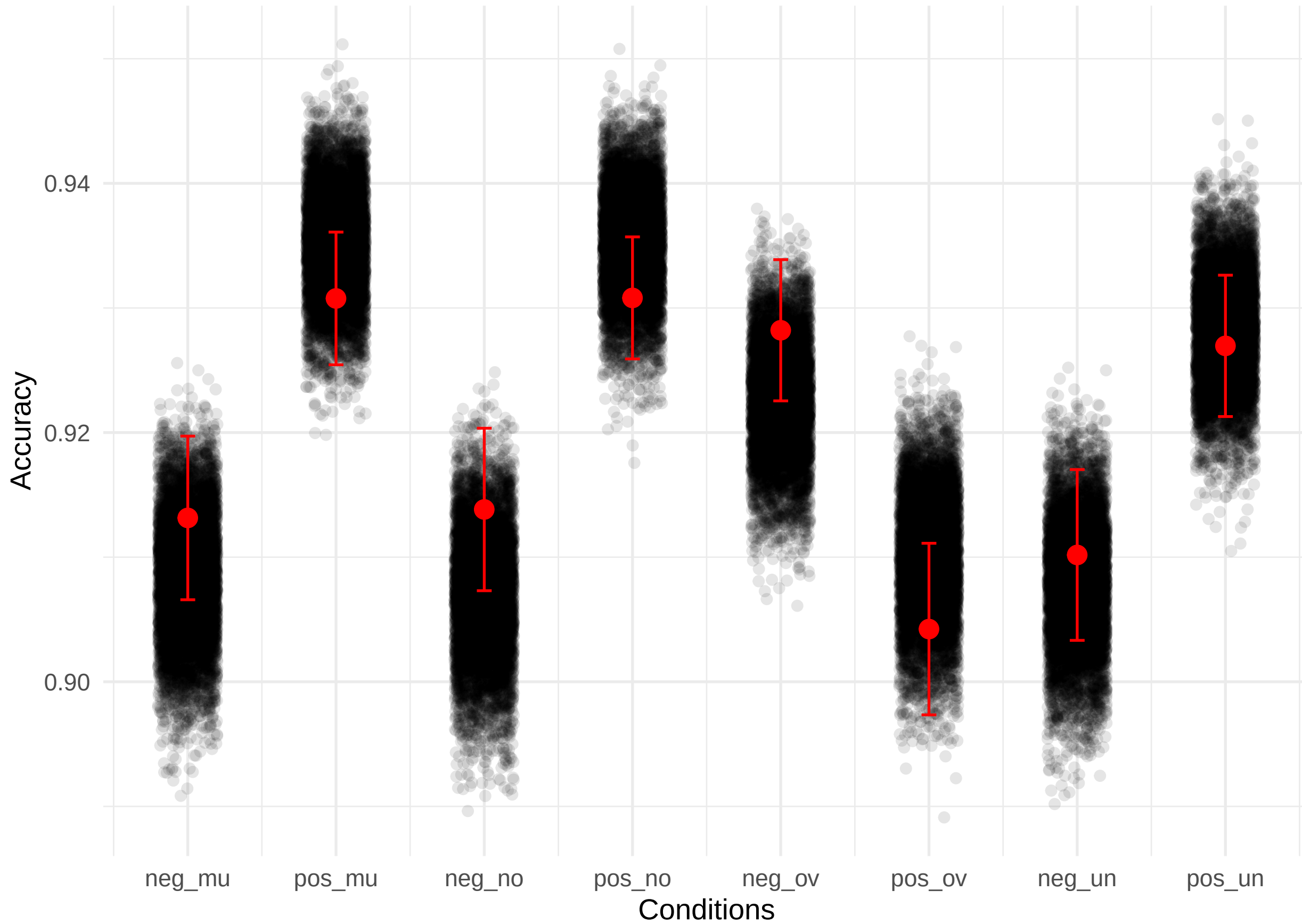

Note. The red points indicate the mean ± 1 standard error of the empirical data. The names of the conditions are structured as follows: the first part refers to the valence of the stimulus (*neg* = negative, *pos* = positive), the second part refers to the prime (*mu* = muscular, *no* = intermediate weight, *ov* = higher weight, *un* = lower weight).

Figure S4

Posterior Predictive of the First Quartile of Response Times for Study 1

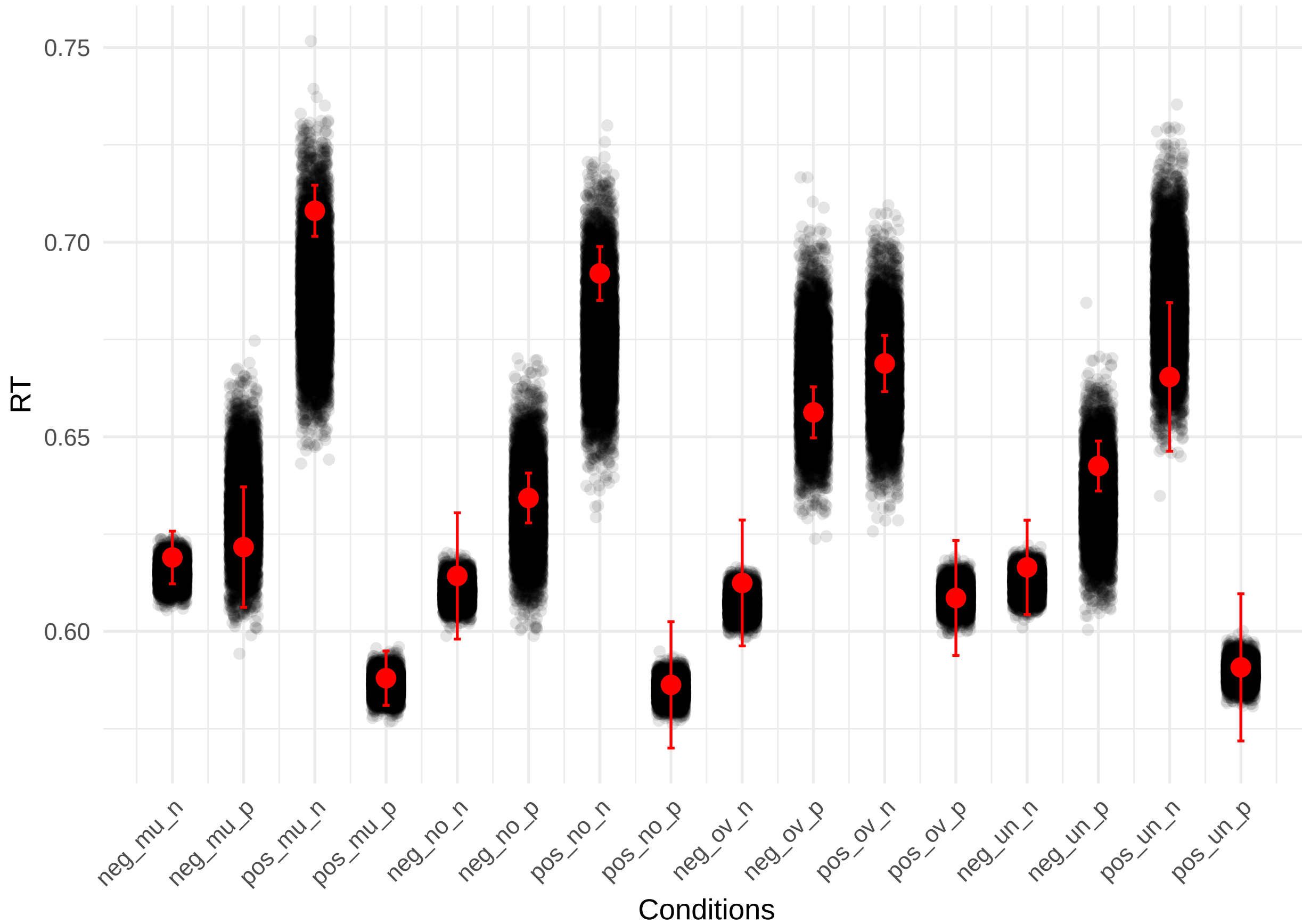

Note. The red point indicates the data. RT = Response Time. The names of the conditions are structured as follows: the first part refers to the valence of the stimulus (*neg* = negative, *pos* = positive), the second part refers to the prime (*mu* = muscular, *no* = intermediate weight, *ov* = higher weight, *un* = lower weight).

Figure S5

Posterior Predictive of the Median of Response Times for Study 1

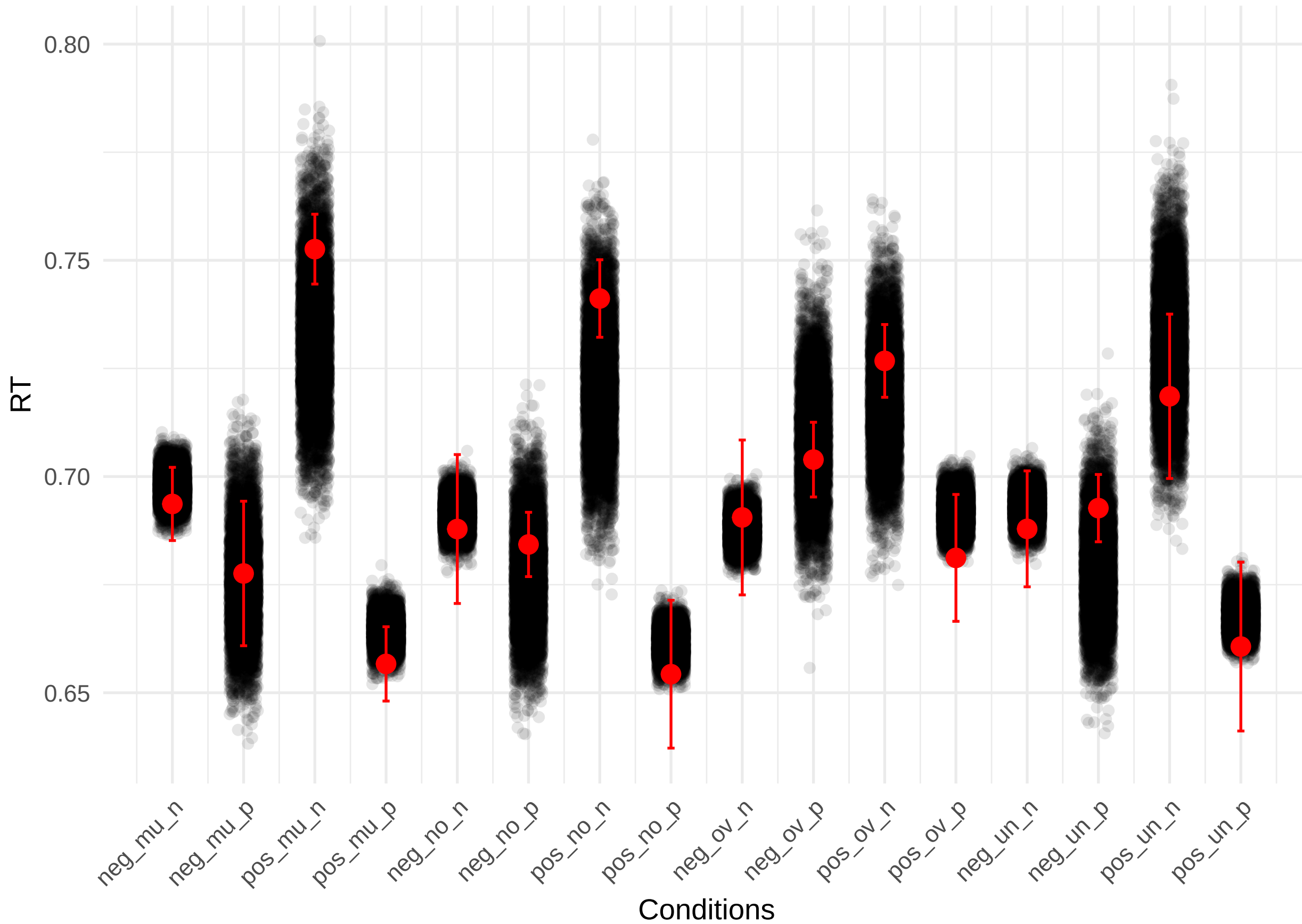

Note. The red points indicate the mean ± 1 standard error of the empirical data. The names of the conditions are structured as follows: the first part refers to the valence of the stimulus (*neg* = negative, *pos* = positive), the second part refers to the prime (*mu* = muscular, *no* = intermediate weight, *ov* = higher weight, *un* = lower weight), the third part to the response of the participant (*n* = negative, *p* = positive).

Figure S6

Posterior Predictive of the Third Quartile of Response Times for Study 1

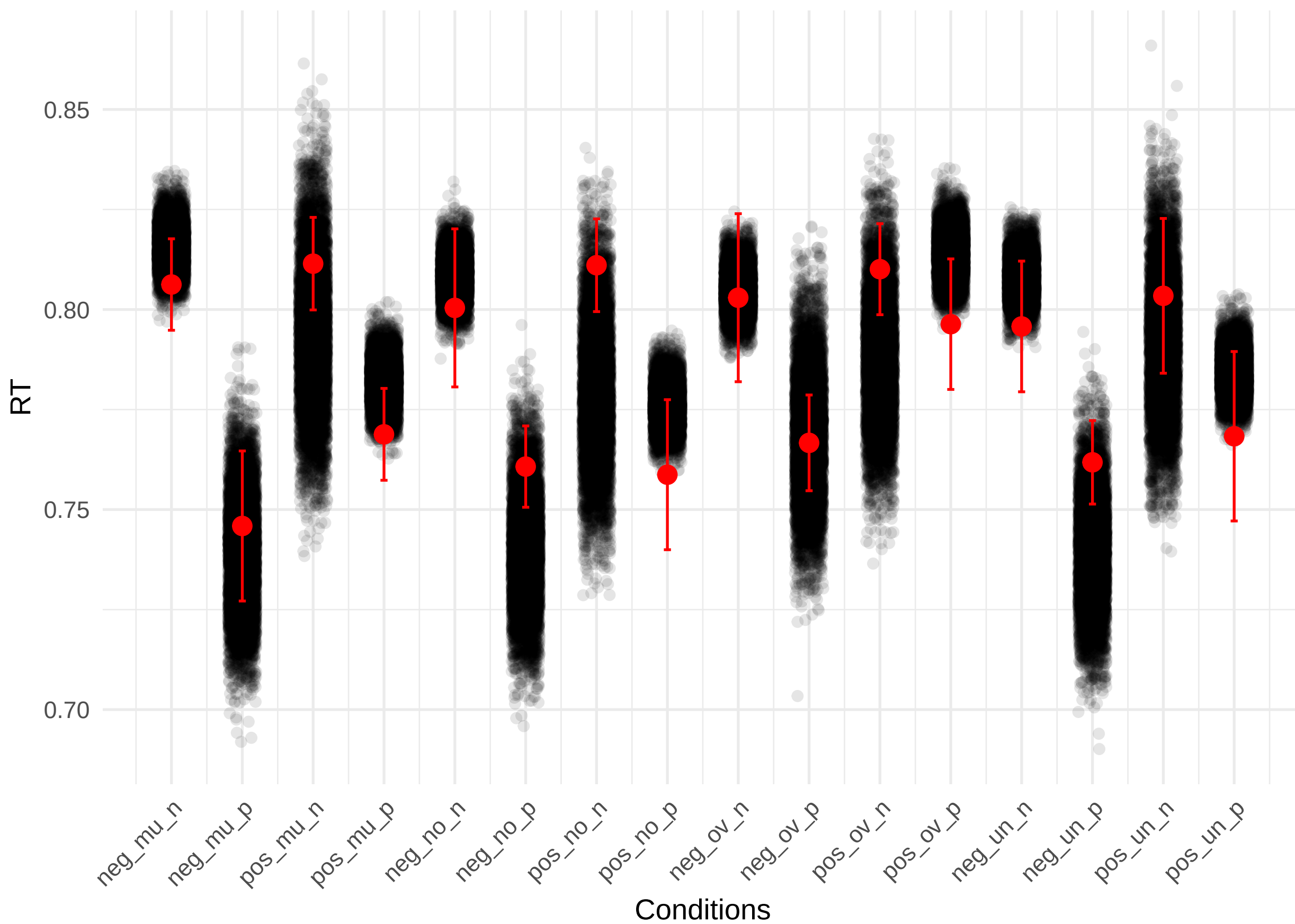

Note. The red points indicate the mean ± 1 standard error of the empirical data. The names of the conditions are structured as follows: the first part refers to the valence of the stimulus (*neg* = negative, *pos* = positive), the second part refers to the prime (*mu* = muscular, *no* = intermediate weight, *ov* = higher weight, *un* = lower weight), the third part to the response of the participant (n = negative, p = positive).

Figure S7

Posterior Distributions of Group-Level Parameters in Study 1

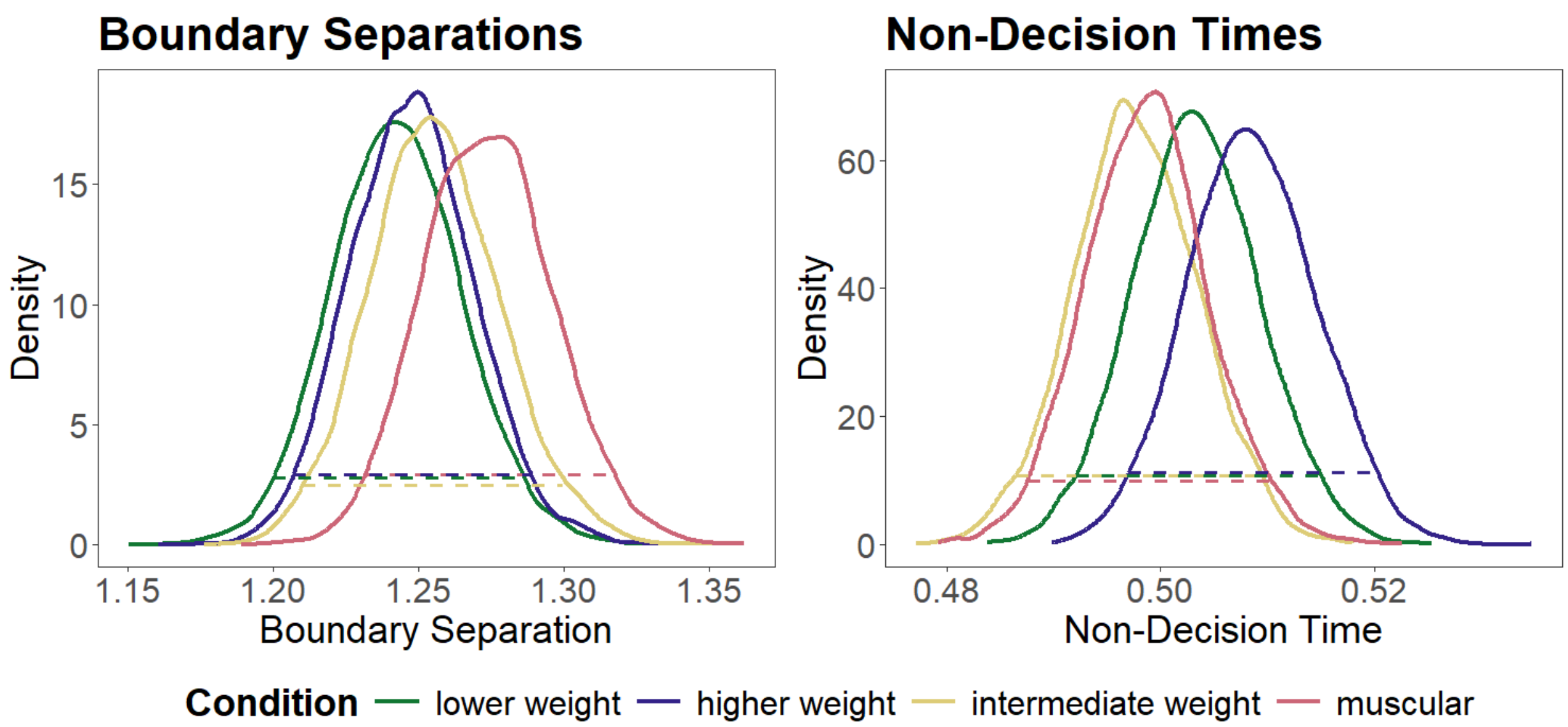

Note. Dashed lines indicate the 95% highest density intervals.

Figure S8

Trace Plots of all Group-Level Parameters for Study 2

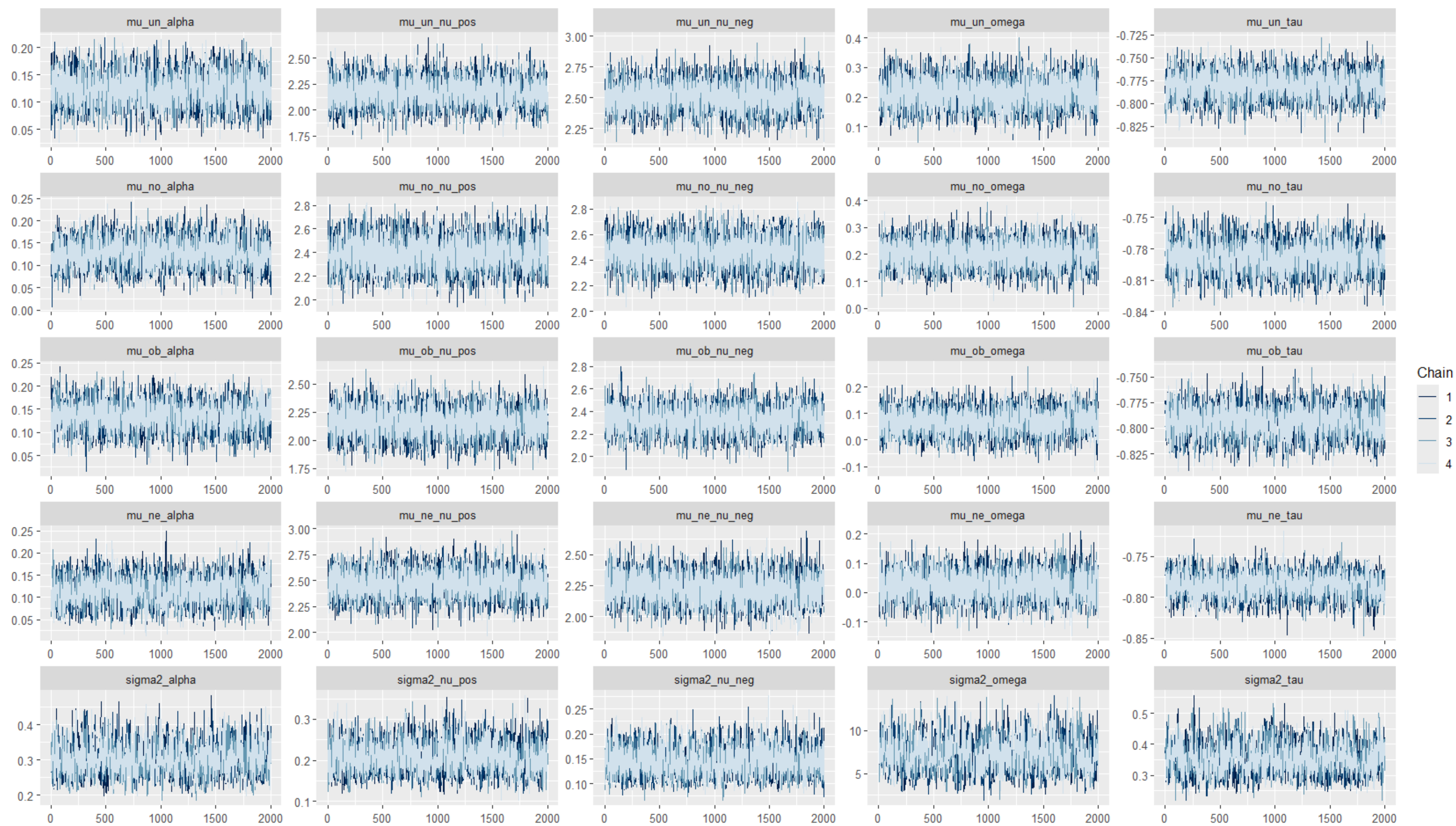

Note. The headings are structured as follows: the first part: mu = mean of group level distribution, sigma2 = variance, the second part (not for sigma): un = lower weight, no = intermediate weight, ob = higher weight, ne = neutral, the third part: alpha = boundary separation, nu = drift rate, omega = starting point, tau = non-decision time, the fourth part (only for drift rates): pos = positive, neg = negative.

Figure S9

Posterior Predictive of the Accuracy Rate for Study 2

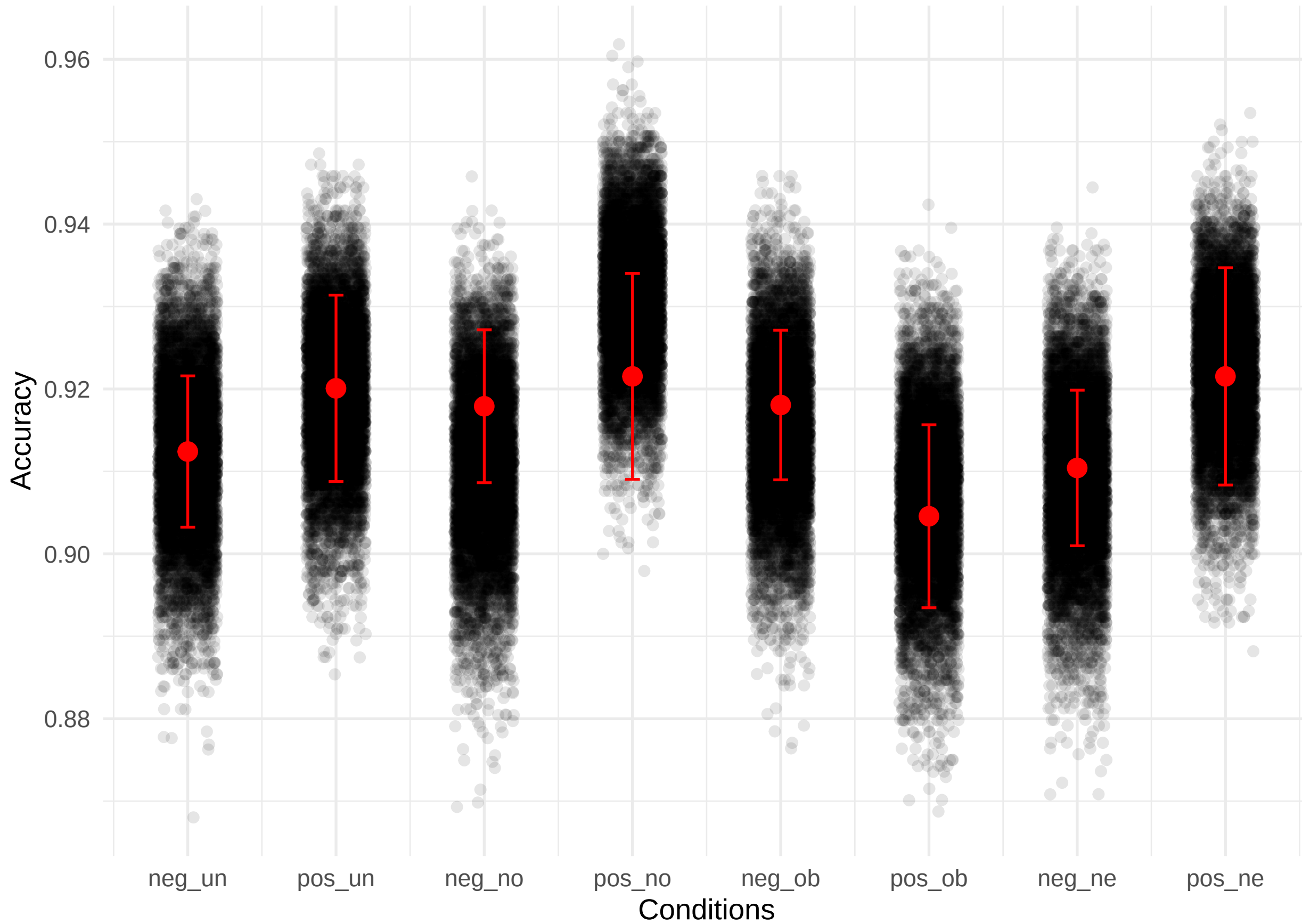

Note. The red points indicate the mean ± 1 standard error of the empirical data. The names of the conditions are structured as follows: the first part refers to the valence of the stimulus (*neg* = negative, *pos* = positive), the second part refers to the prime (*un* = lower weight, *no* = intermediate weight, *ob* = higher weight, *ne* = neutral).

Figure S10

Posterior Predictive of the First Quartile of Response Times for Study 2

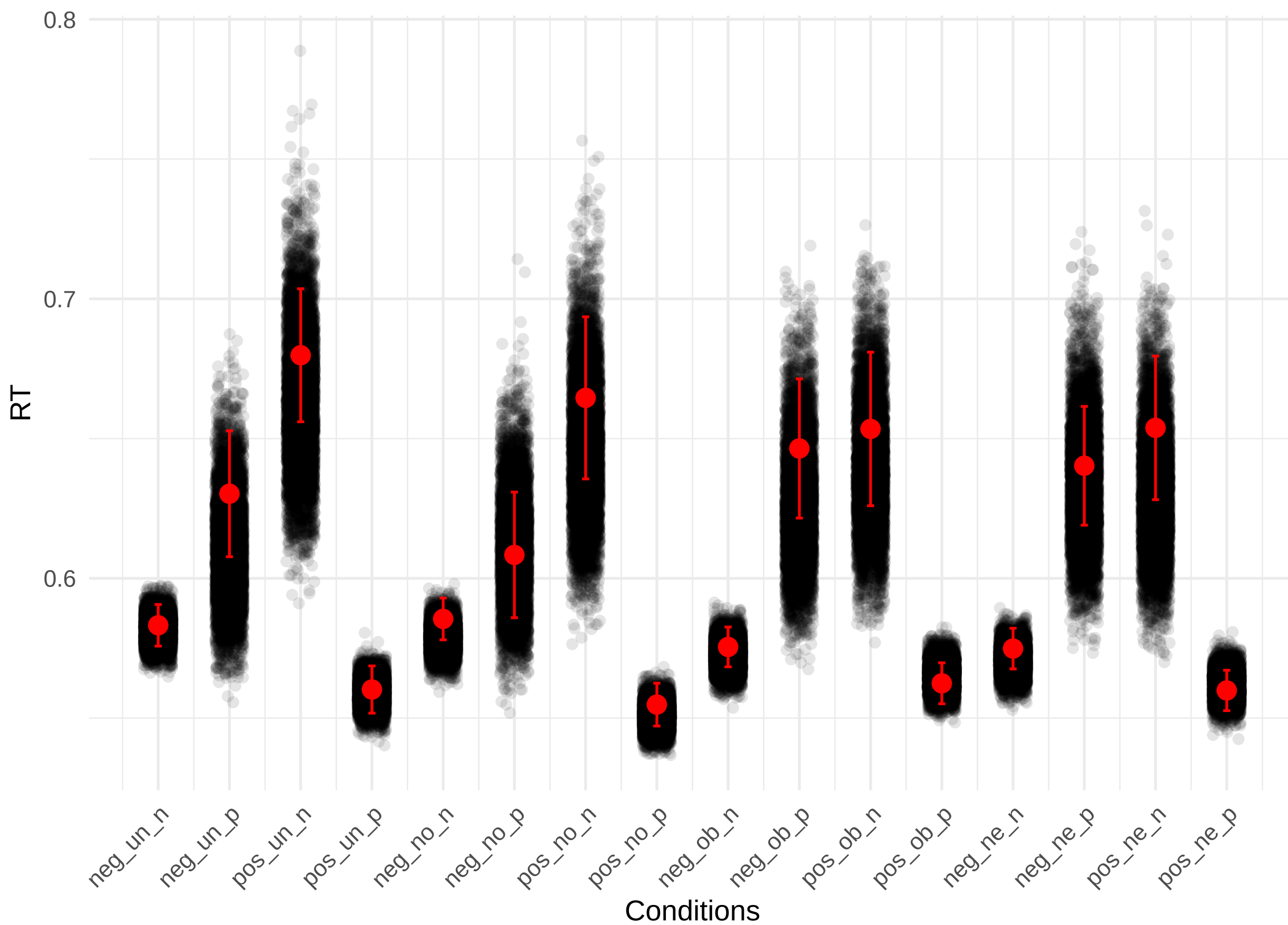

Note. The red points indicate the mean ± 1 standard error of the empirical data. The names of the conditions are structured as follows: the first part refers to the valence of the stimulus (*neg* = negative, *pos* = positive), the second part refers to the prime (*un* = lower weight, *no* = intermediate weight, *ob* = higher weight, *ne* = neutral), the third part to the response of the participant (*n* = negative, *p* = positive).

Figure S11

Posterior Predictive of the Median of Response Times for Study 2

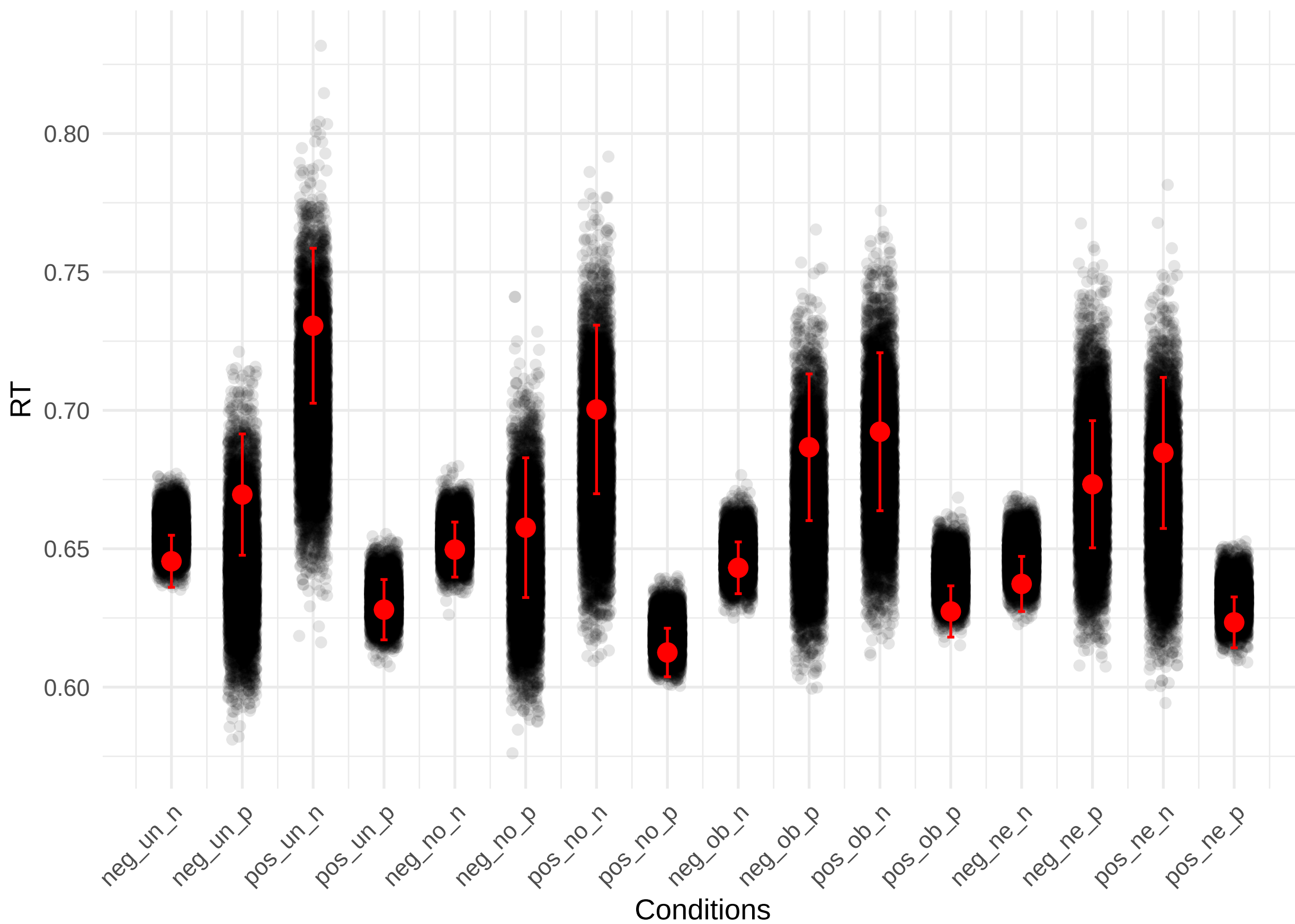

Note. The red points indicate the mean ± 1 standard error of the empirical data. The names of the conditions are structured as follows: the first part refers to the valence of the stimulus (*neg* = negative, *pos* = positive), the second part refers to the prime (*un* = lower weight, *no* = intermediate weight, *ob* = higher weight, *ne* = neutral), the third part to the response of the participant (*n* = negative, *p* = positive).

Figure S12

Posterior Predictive of the Third Quartile of Response Times for Study 2

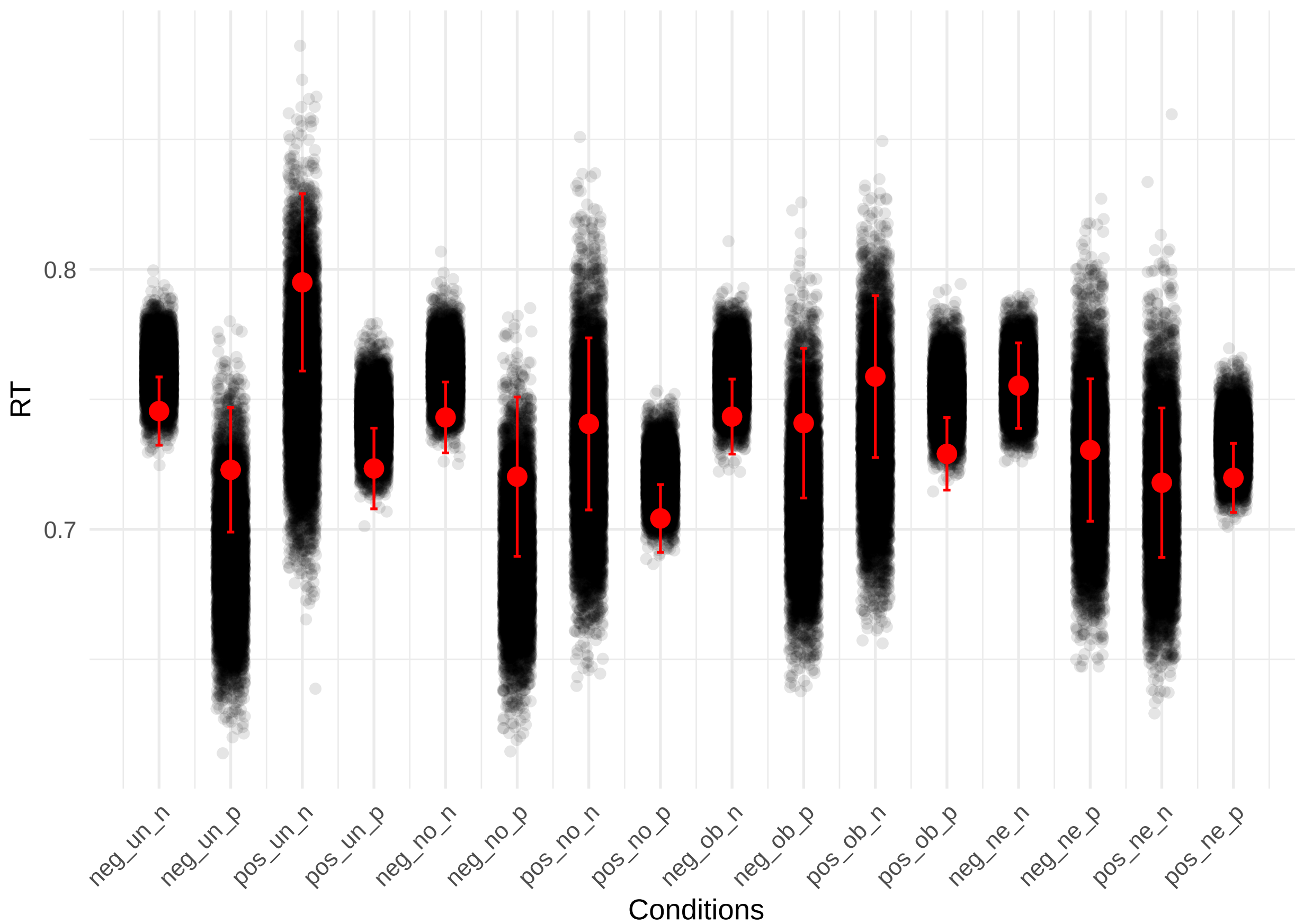

Note. The red points indicate the mean ± 1 standard error of the empirical data. The names of the conditions are structured as follows: the first part refers to the valence of the stimulus (*neg* = negative, *pos* = positive), the second part refers to the prime (*un* = lower weight, *no* = intermediate weight, *ob* = higher weight, *ne* = neutral), the third part to the response of the participant (*n* = negative, *p* = positive).

Figure S13

Posterior Distributions of Group-Level Parameters in Study 2

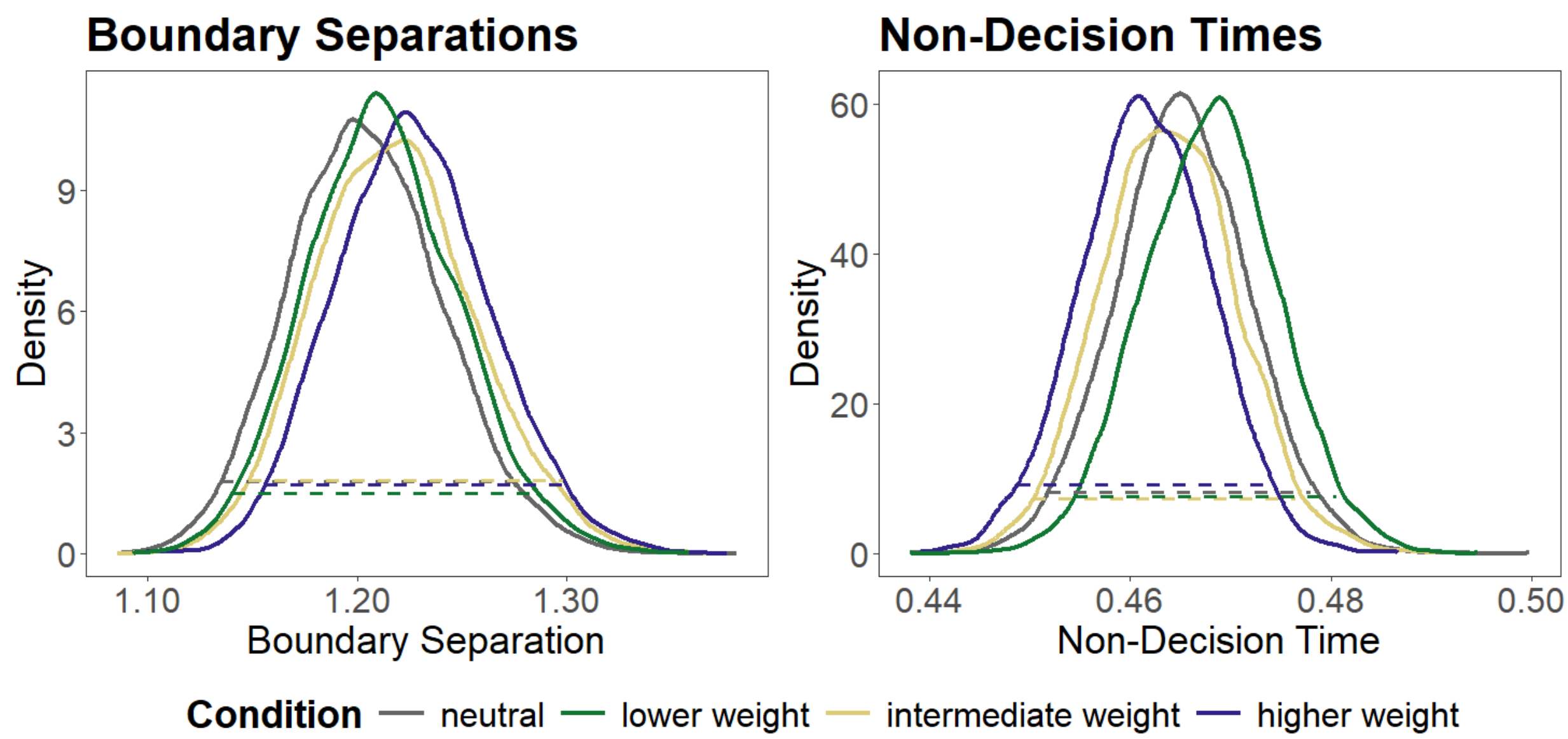

Note. Dashed lines indicate the 95% highest density intervals.
